# A toothed turtle from the Late Jurassic of China and the global biogeographic history of turtles

**DOI:** 10.1186/s12862-016-0762-5

**Published:** 2016-10-28

**Authors:** Walter G. Joyce, Márton Rabi, James M. Clark, Xing Xu

**Affiliations:** 1 Department of Geosciences, University of Fribourg, Chemin de Musée 6, 1700 Fribourg, Switzerland; 2 Department of Geosciences, University of Tübingen, Hölderlinstrasse 12, 72074 Tübingen, Germany; 3 Department of Geosciences, University of Turin, Via Valperga Caluso 35, 10125 Torino, Italy; 4 Department of Biological Sciences, George Washington University, 800 22nd Street, NW, Suite 6000, Washington, DC 20052 USA; 5 Key Laboratory of Vertebrate Evolution and Human Origin of Chinese Academy of Sciences, Institute of Vertebrate Paleontology & Paleoanthropology, Chinese Academy of Sciences, 142 Xizhimenwai Street, Beijing, 100044 China

**Keywords:** Testudinata, Sichuanchelyidae, Helochelydridae, Meiolaniformes, *Sichuanchelys palatodentata*, Jurassic, Xinjiang, China, Phylogeny, Biogeography

## Abstract

**Background:**

Turtles (Testudinata) are a successful lineage of vertebrates with about 350 extant species that inhabit all major oceans and landmasses with tropical to temperate climates. The rich fossil record of turtles documents the adaptation of various sub-lineages to a broad range of habitat preferences, but a synthetic biogeographic model is still lacking for the group.

**Results:**

We herein describe a new species of fossil turtle from the Late Jurassic of Xinjiang, China, *Sichuanchelys palatodentata* sp. nov., that is highly unusual by plesiomorphically exhibiting palatal teeth. Phylogenetic analysis places the Late Jurassic *Sichuanchelys palatodentata* in a clade with the Late Cretaceous *Mongolochelys efremovi* outside crown group Testudines thereby establishing the prolonged presence of a previously unrecognized clade of turtles in Asia, herein named Sichuanchelyidae. In contrast to previous hypotheses, *M. efremovi* and *Kallokibotion bajazidi* are not found within Meiolaniformes, a clade that is here reinterpreted as being restricted to Gondwana.

**Conclusions:**

A revision of the global distribution of fossil and recent turtle reveals that the three primary lineages of derived, aquatic turtles, including the crown, Paracryptodira, Pan-Pleurodira, and Pan-Cryptodira can be traced back to the Middle Jurassic of Euramerica, Gondwana, and Asia, respectively, which resulted from the primary break up of Pangaea at that time. The two primary lineages of Pleurodira, Pan-Pelomedusoides and Pan-Chelidae, can similarly be traced back to the Cretaceous of northern and southern Gondwana, respectively, which were separated from one another by a large desert zone during that time. The primary divergence of crown turtles was therefore driven by vicariance to the primary freshwater aquatic habitat of these lineages. The temporally persistent lineages of basal turtles, Helochelydridae, Meiolaniformes, Sichuanchelyidae, can similarly be traced back to the Late Mesozoic of Euramerica, southern Gondwana, and Asia. Given the ambiguous phylogenetic relationships of these three lineages, it is unclear if their diversification was driven by vicariance as well, or if they display a vicariance-like pattern. The clean, primary signal apparent among early turtles is secondarily obliterated throughout the Late Cretaceous to Recent by extensive dispersal of continental turtles and by multiple invasions of marine habitats.

**Electronic supplementary material:**

The online version of this article (doi:10.1186/s12862-016-0762-5) contains supplementary material, which is available to authorized users.

## Background

Turtles (Testudinata), the clade arising from the first Amniote with a fully formed turtle shell (sensu [1]), currently inhabit all major landmasses with tropical to temperate climates [2]. The clade has an excellent, though often poorly studied fossil record that reaches back to the Late Triassic [3]. Turtles are therefore ideal model organisms to investigate global biogeographic patterns as their evolutionary history coincides with the break-up of the supercontinent Pangaea and the secondary assembly of the continents as seen today. The last 25 years of research using computer assisted cladistic analyses have retrieved an increasingly congruous picture of turtle evolution [4–12] but synthetic biogeographic analyses that include fossil taxa are still rare, noncomprehensive, and either failed to retrieve meaningful global patterns [6, 13] or concentrated on the primary clades of crown-cryptodires [14].

Here we present a new basal turtle, *Sichuanchelys palatodentata* n. sp., from the Late Jurassic of Xinjiang Uygur Autonomous Region, China that is not only unusual for displaying residual palatal teeth, but also has important implications for the global paleobiogeography of turtles. The primary goals of this contribution are therefore to provide a comprehensive description of the new taxon and to re-evaluate the global biogeographic history of the group. The surprising result of this study is that the early evolution of turtles was purely driven by vicariance through the early break-up of Pangaea in the Mesozoic, but that this crisp biogeographic signal was later obscured through profuse dispersal and the invasion of the marine realm.

## Results

### Systematic paleontology

TESTUDINATA Klein, 1760 [15].

SICHUANCHELYIDAE Tong et al., 2012 [16].

*SICHUANCHELYS* Ye and Pi, 1997 [17].

*Sichuanchelys palatodentata* sp. nov.

#### Nomenclatural acts

This published work and the nomenclatural acts it contains have been registered in Zoobank. The LSID for this publication is urn:lsid:zoobank.org:pub:0CB0FE99-2C9E-4953-A688-777D4D71BC37, that of the new species presented herein urn:lsid:zoobank.org:act:B5C8E6B6-C0DA-4BD8-B20F-D9B638AE8491.

#### Etymology

In reference to the presence of palatal teeth. The species epithet is here formed and used explicitly as a noun in apposition and therefore does not have a gender [18].

#### Holotype

**Fig. 1:**
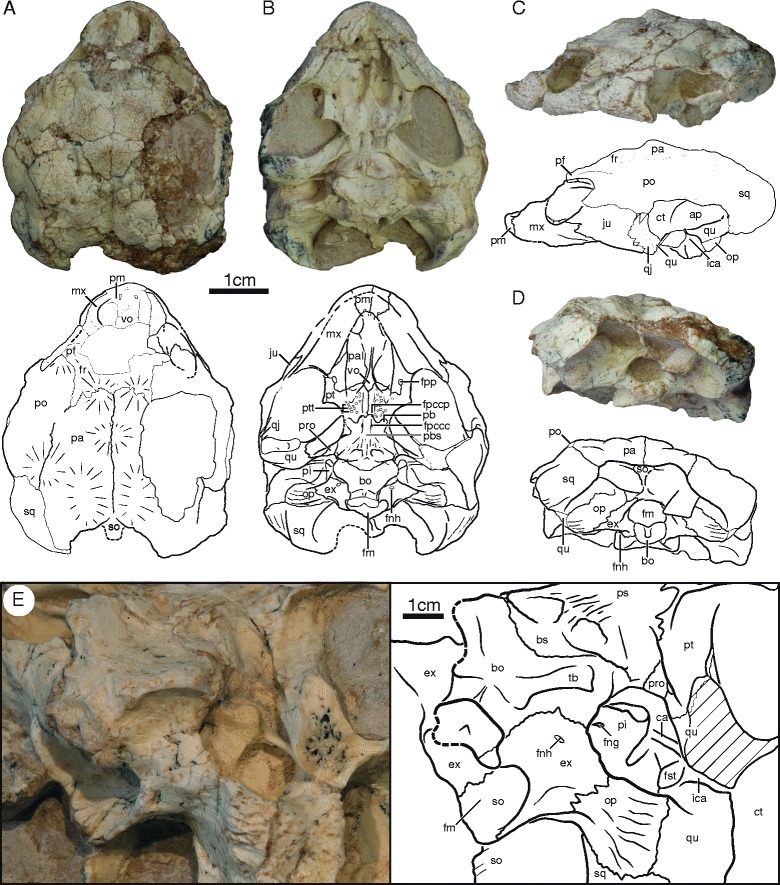
Skull of IVPP V18093, holotype, *Sichuanchelys palatodentata* n. sp., Late Jurassic (Oxfordian), Shishugou Formation, Wucaiwan, Xinjiang, China, in dorsal (**a**), ventral (**b**), left lateral (**c**), posterior (**d**), and oblique view focused on the basicranial region (**e**). *Hatch marks* indicate damaged areas. Abbreviations: ap = antrum postoticum; bo = basioccipital; bs = basisphenoid; ca = columella auris; ct = cavum tympani; ex = exoccipital; fm = foramen magnum; fng = foramen nervi glossopharyngei; fnh = foramen nervi hypoglossi; fpccc = foramen posterius canalis carotici cerebralis; fpccp = foramen posterius canalis carotici palatinum; fpp = foramen palatinum posterius; fr = frontal; fst = foramen stapedio-temporalis; ica = incisura columella auris; ju = jugal; mx = maxilla; op = opisthotic; pa = parietal; pal = palatine; pb = processus basipterygoideus; pbs = parabasisphenoid; pf = prefrontal; pi = processus interfenestralis; pm = premaxilla; po = postorbital; pro = prootic; ps = parasphenoid; pt = pterygoid; ptt = pterygoid teeth; qj = quadratojugal; qu = quadrate; so = supraoccipital; sq = squamosal; tb = tuberculum basioccipitale; vo = vomer

IVPP V18093 (Figs. 1, 3, 4 and 7a), a partial skeleton of a subadult individual consisting of a near complete, slightly crushed skull, missing much of the dorsal skull roofing, complete mandible visible in ventral view, right hyoid, near complete shell lacking the right half of the carapace and most of the pygal region, at least 22 caudals in partial articulation, disarticulated left scapula and coracoid, isolated left pubis, partial right or left manus, including carpals, phalanges, and unguals, left femur, and possible left tibia and fibula. The midline plastron length, excluding epiplastra and entoplastron, is ca. 14 cm. The carapace of this individual is estimated to have had a midline length of ca. 23 cm.

#### Referred material

IVPP V18101–V18103, three poorly preserved carapaces, previously described under the name ?*Sichuanchelys* sp. [19]. Of these, IVPP V18102 is the largest and perhaps corresponds to an adult.

**Fig. 2:**
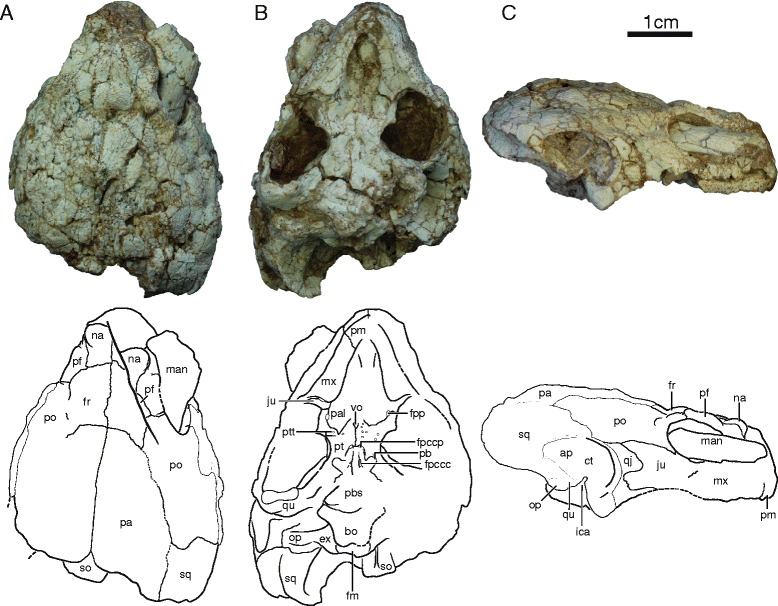
Skull of IVPP V18094, *Sichuanchelys palatodentata* n. sp., Late Jurassic (Oxfordian), Shishugou Formation, Wucaiwan, Xinjiang, China, in dorsal (**a**), ventral (**b**), and right lateral view (**c**). Abbreviations: ap = antrum postoticum; bo = basioccipital; ct = cavum tympani; ex = exoccipital; fm = foramen magnum; fpccc = foramen posterius canalis carotici cerebralis; fpccp = foramen posterius canalis carotici palatinum; fpp = foramen palatinum posterius; fr = frontal; ica = incisura columella auris; ju = jugal; man = mandible; mx = maxilla; na = nasal; op = opisthotic; pa = parietal; pal = palatine; pb = processus basipterygoideus; pbs = parabasisphenoid; pf = prefrontal; pm = premaxilla; po = postorbital; pt = pterygoid; ptt = pterygoid teeth; qj = quadratojugal; qu = quadrate; so = supraoccipital; sq = squamosal; vo = vomer

IVPP V18094 (Figs. 2, 6 and 7b), partial skeleton of a subadult individual that includes a near complete skull crushed along the sagittal axis, left jaw ramus, the damaged anterior plastral lobe, five disarticulated cervical vertebrae, right scapula, crushed left scapulocoracoid, and right humerus. Mid-plastral length, excluding epi- and entoplastron, estimated to be 14 cm by comparison to IVPP V18093.

IVPP V18095 (Fig. 5a), partial skeleton of a subadult consisting of heavily eroded carapace, near complete plastron lacking epiplastra and entoplastron, 2 cervical vertebrae, approximately 20 disarticulated caudals, isolated scapula, and both humeri. Mid-plastral length, excluding epi- and entoplastron, ca. 11 cm.

IVPP V18096 (Figs. 5b and 7c), partial skeleton of a subadult that includes posterior portion of dorsal skull roof, disarticulated carapace consisting of at least 11 costals and five peripherals, near complete plastron lacking the epiplastra and entoplastron, at least two isolated cervicals and two isolated caudals, isolated scapula and ischium, left humerus, femur and tibia, and various unidentified distal limb bones. Mid-plastral length, excluding epiplastra and entoplastron, ca. 11 cm.

IVPP V18097 (not figured), highly fragmentary skeleton of a presumably adult individual consisting of heavily fragmented, partial skull comprised of nasal region, left cheek region, both prootics and partial quadrates, and partial basisphenoid, basioccipital, and exoccipital, near complete, but fragmented mandible, left lateral third of shell missing the nuchal and pygal region, at least two isolated caudals, and various unidentifiable fragments of long bones. Mid-plastral length, excluding epiplastra and entoplastron, ca. 20–25 cm.

#### Locality and horizon

All specimens herein referred to the new taxon were collected from the early Upper Jurassic (Oxfordian) upper part of the Shishugou Formation at the Wucaiwan Locality in Xinjiang Uygur Autonomous Region, China (see [20] for map). The holotype and referred specimens IVPP V18094–18096 (see below) were found in close association to one another, along with nearly complete, articulated skeletons of a squamate and a shartegosuchid crocodyliform. IVPP V18097 was recovered 1.2 km to the north of the type locality and IVPP V18102 an additional 2.2 km northward. The type locality is positioned between two tuffs (T-2 and T-BW of [20]) and can thereby be dated securely to the early Oxfordian. V18102 and V18097 were recovered from sediments slightly higher in the formation, just above the T-BW tuff dated 159.7+/-0.3 million years ago, but still thought to be Oxfordian in age considering locally calculated sedimentation rates. Precise locality information is unavailable for V18101 and V18103 within Wucaiwan, but they are likely from the upper part of the Shishugou Formation, and therefore Oxfordian as well.

#### Diagnosis

*Sichuanchelys palatodentata* sp. nov. can be diagnosed as a representative of *Sichuanchelys* by the following, unique combination of shell characters: broad nuchal emargination delimited by peripheral II, vertebral scutes broader than long, marginal restricted to peripherals, ligamentous bridge, broad plastron, one pair of fully developed mesoplastra, short midline contact of epiplastra, and anteroposteriorly short extragular scutes. *Sichuanchelys palatodentata* is differentiated from *S. chowi* by a consistent contact of vertebral I with marginal II, by being larger, and, perhaps, by retaining a central plastral fontanelle when reaching maturity. Using cranial characters, *S. palatodentata* can be diagnosed to be closer to extant turtles than *Proganochelys quenstedti* Baur, 1887 [21] by having a fused basicranial joint, an anteriorly shifted canalis stapedio-temporalis, and by lacking vomerine and palatine teeth, but more basal relative to extant turtles by possessing pterygoid teeth, visible remnants of the basipterygoid process, and a prootic that is visible in ventral view. *Sichuanchelys palatodentata* can be distinguished from *Kayentachelys aprix* Gaffney et al., 1987 [22], the only other known turtle with this combination of three plesiomorphic characters, by the exclusion of the frontals from the orbit, an elongated jugal that nearly contacts the quadrate posteriorly, posteriorly extended squamosals, and a closed interpterygoid vacuity and formed foramina posterius canalis carotici palatinum.

#### Comments

The new material we present herein from the Late Jurassic of Xinjiang Province overlaps in overall shape and shell texture with that of previously described material *Sichuanchelys chowi* from the Middle Jurassic of Sichuan Province and it is therefore not surprising that initial finds from Xinjiang were identified as *Sichuanchelys* sp. [19]. The available material of *Sichuanchelys chowi* only consists of shells [16] and has not yet been described in detail. We are therefore not able to make detailed comparisons, although sufficient insights are available to distinguish a new taxon. Given the lack of character information for *S. chowi*, our phylogenetic analysis (see Methods below) is furthermore not able to rigorously resolve the relationships within Sichuanchelyidae. As we do not favor naming a new genus based on poor character evidence, we here place the new species in *Sichuanchelys*, but note that future analyses may not resolve this taxon to be monophyletic.

#### Phylogenetic nomenclature

We generally follow previously established phylogenetic nomenclature [23–26]. In addition, we herein phylogenetically redefine the name Sichuanchelyidae Tong et al., 2012 [16] as referring to the clade that includes all turtles more closely related to *Sichuanchelys chowi* Ye and Pi, 1997 [17] than to *Meiolania platyceps* Owen, 1886 [27], *Helochelydra nopcsai* Lapparent de Broin and Murelaga, 1999 [28], or any extant turtle. The name Mongolochelyidae “Sukhanov and Pozdnjakov, In Press” (as provided in [29]) is not used herein, because Sukhanov and Pozdnjakov, In Press never appeared in print and because the name was otherwise never formally designated as a new family group taxon [30–32]. This name is therefore not available for nomenclatural consideration [18]. We similarly define the name Helochelydridae Nopcsa 1928 [33] as referring to the clade that includes all turtles more closely related to *Helochelydra nopcsai* than to *Meiolania platyceps*, *Sichuanchelys chowi*, or any extant turtle. The rule of priority also applies to names within the family group [18] and we therefore disregard Solemydidae Lapparent de Broin and Murelaga 1996 [34] since it is the junior synonym of Helochelydridae.

### Description

#### Skull

We herein utilize previously established terminology for cranial anatomy [35] with recent amendments [11] in regards to the carotid circulation.

At least four skulls are present in varying degrees of preservation. The skull of IVPP V18093 shows the least amount of distortion, particularly in ventral view, but much of the dorsal surface is missing and crushed (Fig. 1). The skull of IVPP V18094 is the most complete, but the surface is heavily fractured and the shape is greatly distorted by shearing. In particular, the right orbital region is shifted to the posterior, the left ear is deformed, and the entire skull is slightly crushed along the sagittal plane (Fig. 2). Only the posterior margin of the skull roof is preserved in IVPP V18096 and only fragments are associated with IVPP V18097 (not figured). This description is therefore based mostly on IVPP V18093 and IVPP V18094.

The skull is relatively low, the external nares are confluent, the orbits face laterally, and there are no signs of temporal emargination, lacrimals, or supratemporals. The parietals and squamosals form an incipient ‘collar’ (i.e., a posterior expansion of the dorsal skull roof) that protrudes posterior beyond the regular margin of the skull. The entire skull roof is decorated by numerous protuberances that we interpret as evidence for cranial scales, but well-defined sulci are not apparent. The frontals, parietals, and postorbitals combined are decorated by at least one unpaired and four paired scales (Fig. 1a), but we refrain from homologizing them until the skull roof is better understood for this taxon. Particularly well-developed horn-like protuberances are present around the dorsal margin of the orbit.

##### Nasal

The nasal is a relatively large, rectangular element that forms the dorsal margin of the external nares and contacts the maxilla ventrally, the prefrontals posterolaterally, the frontal posteriorly, and its counterpart medially (Fig. 2). The anterior margin is decorated by a prominent, bulbous protrusion. The full outline of the external nares is not preserved in any specimen, but the well-preserved ventral margin of IVPP V18093 demonstrates that the external nares were not subdivided by the premaxillae.

##### Prefrontal

The dorsal plate of the prefrontal is slightly smaller than that of the nasal. The prefrontal forms the anterodorsal portion of the rim of the orbit and contacts the maxilla ventrally, the nasal anteromedially, the frontal posteromedially, and the postorbital posteriorly (Figs. 1 and 2). The dorsal plate is decorated by bulbous protrusions, particularly along the margin of the orbit. The descending plate of the prefrontal forms the anterior wall of the orbit and contacts the vomer, palatine, and maxilla ventrally. The foramen orbito-nasale is located at the contact between the prefrontal, vomer, and maxilla and is reduced to the size of a pinhole. The outline of the sulcus olfactorius is not preserved.

##### Frontal

The frontal is a sub-triangular element that does not contribute to the orbit and that contacts the nasals along a slightly oblique suture anteriorly, the prefrontal anterolaterally, the postorbital laterally, the parietal along a heavily interdigitated suture posteriorly, and its counterpart medially (Figs. 1 and 2).

##### Parietal

The parietal is the largest element on the dorsal skull roof. It contacts the frontal anteriorly, the postorbital anterolaterally, and squamosal posterolaterally, the supraoccipital posteriorly, and its counterpart along the midline (Figs. 1 and 2). The parietals combined form a midline scale protrusion in their anterior third, a pair of anterior scale protrusions together with the frontals, a pair of posterior anteroposteriorly elongate scale protrusions in their posterior third, and a pair of scale protrusions along the suture with the postorbital. The inferior process of the parietal is not preserved in any specimen and its extent and possible contacts are therefore not known.

##### Jugal

The anterior portion of the lateral plate of the jugal is best preserved in IVPP V18093, whereas the posterior portion is best preserved in IVPP V18094. The jugal forms the posteroventral rim of the orbit, contacts the maxilla anteroventrally, the postorbital anterodorsally, and the quadratojugal posteriorly (Figs. 1 and 2). The posterior portion of the jugal is split into ventral and dorsal processes that surround much of the lateral exposure of the quadratojugal. The ventral process nearly contacts the quadrate. The medial plate of the jugal contacts the maxilla and palatine within the orbit and additionally contacts the pterygoid within the lower temporal fossa.

##### Quadratojugal

The lateral exposure of the quadratojugal is greatly reduced by the jugal (Figs. 1 and 2). The quadratojugal contacts the jugal anteriorly and frame the anterior rim of the cavum tympani.

##### Squamosal

The dorsal exposure of the squamosal contacts the postorbital anteriorly, the parietal medially, and frames the posterodorsal portion of the cavum tympani together with the quadrate (Figs. 1 and 2). The squamosals form distinct posteromedial protrusions that form a ‘collar’ together with the parietals that is intermediate between the condition seen in most turtles and the extreme collar apparent in *Mongolochelys efremovi*. In posterior view, the squamosal broadly contacts the paroccipital process of the opisthotic. The posterolateral aspects of the squamosal are decorated by fine striations.

##### Postorbital

The dorsal exposure of the postorbital is only slightly smaller than that of the parietal (Figs. 1 and 2). The postorbital forms the posterior margin of the orbit, contacts the prefrontal and frontal anterolaterally, broadly contacts the parietal medially, the squamosal posteriorly, and the jugal, quadratojugal, and quadrate ventrolaterally. The dorsal surface is decorated by a distinct scale protrusion along the posteromedial rim of the orbit and a broad scale protrusion along the posteromedial contact with the parietal. A descending process is absent.

##### Premaxilla

The premaxillae are paired and contribute to the ventral margin of the external nares and the anterior portion of the labial ridge and the triturating surface (Figs. 1 and 2). The premaxilla contacts the maxilla posterolaterally and the vomer posteriorly. A pair of small prepalatine foramina pierce the premaxilla in dorsal view at mid-length, but exit in ventral view at the contact with the vomer.

##### Maxilla

In lateral view, the maxilla forms the anteroventral aspects of the orbit, and contacts the premaxilla anteriorly, the nasal and prefrontal dorsally, and the jugal posteriorly (Figs. 1 and 2). Within the orbit, the maxilla contacts the prefrontal anteriorly, the palatine medially, and the jugal posteriorly. In ventral view, the maxilla forms the majority of the lingual margin and the relatively broad, flat triturating surface and contacts the premaxilla anteromedially, the palatine medially, and the jugal posteriorly. The labial ridge is straight in lateral view and the palatine and jugal do not contribute to the triturating surface. A small contact with the vomer is perhaps present just posterior to the contact with the premaxilla.

##### Vomer

The vomer is an elongate, toothless, and unpaired element best preserved in IVPP V18093 (Fig. 1b). The anterior third of this bone is flat and unusually wide, the intermediate third is decorated by a distinct ridge, and the posterior third is narrow and flat. The vomer contacts the premaxilla anteriorly, the palatine laterally, and the pterygoid posteriorly. A minute anterolateral contact may perhaps be present with the maxilla. An anterolateral contact is apparent with the prefrontal in dorsal view.

##### Palatine

The palatine is a flat element that lacks teeth, forms much of the roof of the primary palate, and frames the internal narial opening anteriorly and contributes to the medial aspects of the small foramen palatinum posterius (Figs. 1 and 2). The palatine contacts the vomer medially, the pterygoid posteriorly, and the maxilla laterally. In dorsal view, the palatine forms much of the floor of the orbit and contacts the prefrontal anteriorly, the maxilla anterolaterally, and the jugal posterolaterally.

##### Quadrate

In lateral view, the quadrate forms a well-developed, kidney-shaped cavum tympani and contacts the quadratojugal anteriorly, the postorbital anterodorsally, and the squamosal posterodorsally (Figs. 1 and 2). The region posterior to the incisura columella auris is greatly inflated. In most turtles this area is laterally covered by bone to form the antrum postoticum, but in *Sichuanchelys palatodentata* most of this cavity remains laterally open. This condition is otherwise only seen in *Mongolochelys efremovi*. The quadrate does not fully encircle the anterior opening of the antrum, instead the dorsal portion is formed by the squamosal. The incisura columella is clearly incised into the posterior aspect of the quadrate, but remains open towards the posterior. The articular processes are low and face anteroventrally.

In ventral view, the quadrate contacts the pterygoid medial to the articular processes. Posterior to the incisura columella auris, the quadrate has a broad posteromedial contact with the distinct paroccipital process of the opisthotic and a posterior contact with the squamosal. The anteromedial contacts of the quadrate within the upper temporal fossa are not visible in available specimens.

IVPP V18097 is the only specimen to partially preserve the quadrate in dorsal view. The specimen is too fragmentary to demonstrate the presence of a processus trochlearis oticum, but the dorsal surface of the quadrate is nevertheless decorated by a roughened surface similar to that developed in *Mongolochelys efremovi*. This roughened surface likely indicates the former presence of a cartilaginous cap in this region and can be seen as evidence of an otic trochlear mechanism [8].

##### Epipterygoid

The epipterygoids, if present, are fully obscured by sediment and their morphology therefore cannot be discerned.

##### Pterygoid

The ventral surface of each pterygoid in IVPP V18093 is decorated by about a dozen circular structures that are arranged in a V-shaped pattern with its apex posteriorly (Figs. 1 and 2). About half of these structures consist of a ring of dense tissue and can therefore be interpreted safely as palatal teeth. All but one of these teeth broke near their base. The remaining circular structures are shallow depressions lacking any evidence of enamel and therefore represent facets from which palatal teeth dislodged either pre or post mortem. The ventral surfaces of the pterygoids of IVPP V18094 are poorly preserved, but a number of pterygoid teeth can be identified here as well. There is no evidence for palatal teeth on the vomer and the palatines.

The anterior branch of the pterygoid has a small contact with the jugal anterolaterally and with the vomer anteromedially. In addition, it contributes to the lateral margin of the foramen palatinum posterius, contacts the palatine anteriorly, and broadly contacts its counterpart along the midline. The external pterygoid process is clearly developed, has a small posterior projection, and a small, but distinct vertical plate. The pterygoid has a short, but clear sutural contact with the parabasisphenoid (sensu [36]) along the midline. The interpterygoid vacuity is therefore closed and the palatine artery enters the skull through a distinct foramen posterius canalis carotici palatinum formed by the pterygoid and the basisphenoid. The pterygoid furthermore has a sutural articulation with the parabasisphenoid posterior to the basipterygoid process and fully surrounds the basipterygoid process. The posterior half of the pterygoid contacts the prootic posteriorly and the quadrate posterolaterally and only partially floors the cranio-quadrate space. The prootic therefore remains exposed in ventral view (Fig. 1e). Much of the cavum acustico-jugulare is open in ventral view due to the short posterior process of the pterygoid failing to reach the basioccipital or the exoccipital. The quadrate process forms a thin lamina of bone that partially floors the incipient canalis cavernosus in ventral view.

##### Supraoccipital

In posterior view, the supraoccipital forms the dorsal margin of the foramen magnum and contacts the exoccipitals ventrally (Figs. 1 and 2). The supraoccipital crista is short and likely did not protrude significantly beyond the level of the occipital process. The distal tip of the crista is expanded into a horizontal shelf that is partially visible in dorsal view behind the parietals. However, the shelf does not contribute directly to the dorsal roofing of the skull. The ventrolateral contacts of the supraoccipital within the upper temporal fossa are not preserved, beyond the posterolateral contact with the opisthotic.

##### Exoccipital

The exoccipital forms the lateral margin of the foramen magnum, contacts the supraoccipital dorsally, the opisthotic laterally, and the basioccipital medioventrally (Figs. 1 and 2). The occipital condyle is damaged in all specimens, but it is nevertheless apparent that the exoccipital contributed to the dorsolateral portion of the condyle. The exoccipital forms a bony wall that defines the posterior border of the recessus scalae tympani and that is pierced by a single hypoglossal foramen, which is oriented slightly to the anterior and thereby easily overlooked in posterior view. A notch at the lateral margin of this wall may either be a second, partially developed hypoglossal foramen or a partially developed posterior jugular foramen.

##### Basioccipital

The basioccipital contacts the basisphenoid anteriorly along a deeply concave suture, the exoccipital dorsally, and forms the ventral rim of the foramen magnum (Figs. 1 and 2). The occipital condyle is damaged in all specimens, but it is apparent that it is situated dorsal to the ventral surface of the basioccipital and that the basioccipital forms the central portion of the process. The ventral surface of the basioccipital forms a broad depression together with the posterior portion of the basisphenoid that is defined laterally by a single pair of distinct tubercles.

##### Prootic

The prootic is only partially covered by the pterygoid ventrally and is therefore visible in ventral view, where it contacts the pterygoid laterally, the parabasisphenoid medially, and roofs the incipient canalis cavernosus (Figs. 1 and 2). Matrix and the dorsal skull roof obscure the contacts of the prootic within the temporal fossa in IVPP V18093 and V18094. In IVPP V18097 the prootic contacts the quadrate laterally and ventrally, contributes to the foramen stapedio-temporale and the trigeminal foramen, but not to the trochlear process.

##### Opisthotic

The paroccipital process is a conspicuous, vertically oriented extension of the opisthotic that has a sutural articulation with the quadrate and squamosal laterally, is decorated distally by fine striations, and is partially visible in lateral view (Figs. 1 and 2). In posterior view, the opisthotic otherwise has a broad dorsolateral contact with the squamosal, and broad ventromedial contact with the exoccipital, and a short lateral contact with the supraoccipital. Within the upper temporal fossa, the opisthotic contacts the quadrate laterally and the supraoccipital medially, but a possible contribution to the margin of the foramen stapedio-temporale is obscured in all skulls. The processus interfenestralis is exposed in ventral view in the form of a well-developed, slender process that is oriented anteroventrally. It has a ventral expansion, and possibly contacts the basioccipital, but does not contribute to the ventral surface of the skull. The processus interfenestralis forms the posterior rim of the fenestra ovalis, forms the anterior wall of the recessus scalae tympani, and constricts the perilymphatic fenestra to the size of the foramen nervi hypoglossi.

##### Parabasisphenoid

The parabasisphenoid consists of the basisphenoid and the parasphenoid [36]. Its dual composition is best revealed at its posterior end, where it is possible to discern the parasphenoid as a thin lamina that only partially overlaps the basisphenoid in this region (Fig. 1e).

The anterior half of the parabasisphenoid forms a broad and rounded midline ridge (Figs. 1 and 2). This ridge has a sutural contact with the pterygoid anteriorly, thereby fully closing the medial portion of the interpterygoid vacuity. The remaining, lateral portions of the interpterygoid vacuity are reduced to form a pair of foramina posterius canalis carotici palatinum, which are situated on both sides of the elevated midline ridge. Further to the posterior, a pair of foramina posterius canalis carotici cerebralis pierces the parabasisphenoid at the level of the basipterygoid process, again along the sides of the elevated midline ridge. The basipterygoid processes of the parabasisphenoid are distinct, rounded lobes that are oriented in a ventrolateral angle and that are firmly sutured to the pterygoid.

The posterior half of the parabasisphenoid is significantly broader than the anterior half and has a sutural contact with the prootic laterally and a posterior convex contact with the basioccipital posteriorly (Figs. 1 and 2). The central ridge of the anterior half expands along the posterior half of the parabasisphenoid to cover a triangular area that spans nearly the full breadth of the bone and that is punctured by a pair of large, deep pits. Coarse, anteroposteriorly-oriented ridges decorate all elevated portions of the triangular area. There is no sign of a vidian foramen on the ventral surface of the parabasisphenoid. The parabasisphenoid of *Sichuanchelys palatodentata* resembles that of *Mongolochelys efremovi* in its bony contacts, the development of the posteriorly expanded and ventrally decorated medial ridge, the placement of the carotid foramina, the presence of a pair of pits, and the presence of clearly developed basipterygoid processes.

##### Columella Auris

The columellae auris are preserved on both sides of the skull in IVPP V18093, but both ends still remain in matrix (Figs. 1 and 2). It is nevertheless clear that the columella auris is a slender element that fills the fenestra ovalis medially and is attached to the tympanic membrane laterally.

##### Mandible

**Fig. 3:**
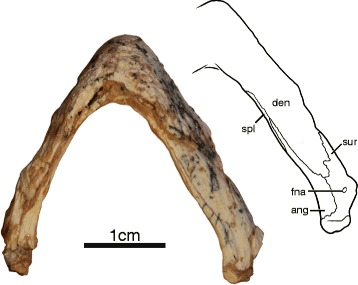
Mandible of IVPP V18093, holotype, *Sichuanchelys palatodentata* n. sp., Late Jurassic (Oxfordian), Shishugou Formation at Wucaiwan, Xinjiang, China, in ventral view. Abbreviations: ang = angular; den = dentary; fna = foramen nervi auriculotemporalis; spl = splenial; sur = surangular

IVPP V18093 and IVPP V18094 are the only specimens to preserve their mandible, however, only the ventral side of the left ramus of IVPP V18093 is well preserved, whereas the rest remains embedded in sediment (Fig. 3). The mandibular rami are relatively narrow and lack expanded triturating surfaces. The dentaries are fused along the midline and form the majority of the mandible. The dentaries contact the surangulars posteriorly along an interdigitated suture, the angulars posteroventrally, and the splenials ventrally. The splenial is an elongate, flat element that forms the ventromedial aspects of the rami. It has an elongate posteromedial contact with the angular and an elongate lateral contact with the dentary that closely approaches the symphysis. The surangular is pierced by a small foramen nervi auriculotemporalis. The articulars cap the posterior ends of the rami. The lateral view of the left ramus of IVPP V18094 reveals that the lower jaws were massive relative to the slender ones of coeval xinjiangchelyids [11, 19, 25] and that the coronoid process was relatively high. The right ramus of this specimen is lodged into the orbit and does not reveal any additional details.

#### Carapace

**Fig. 4:**
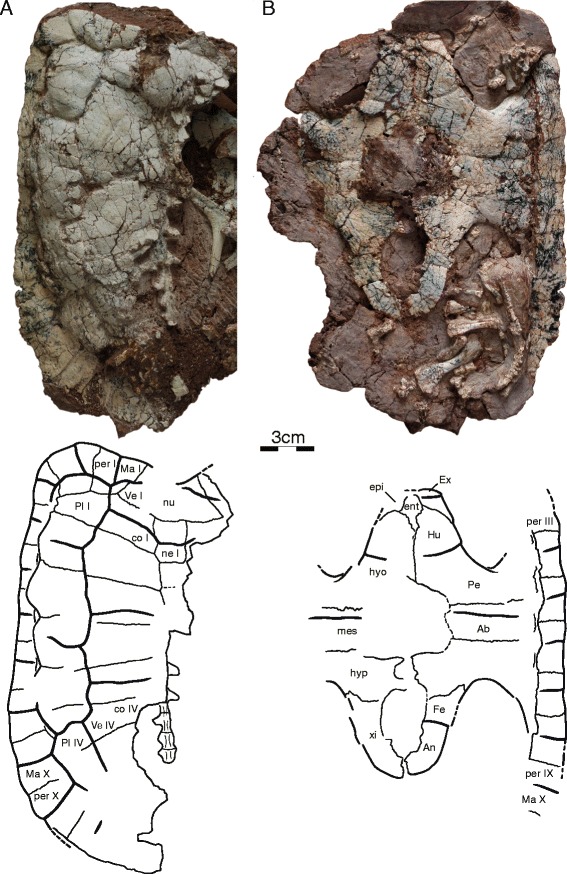
Shell of IVPP V18093, holotype, *Sichuanchelys palatodentata* n. sp., Late Jurassic (Oxfordian), Shishugou Formation, Wucaiwan, Xinjiang, China, in dorsal (**a**) and ventral (**b**) view. Abbreviations: Ab = abdominal scute; An = anal scute; co = costal; ent = entoplastron; epi = epiplastron; Ex = extragular scute; Fe = femoral scute; Hu = humeral scute; hyo = hyoplastron; hyp = hypoplastron; Ma = marginal scute; mes = mesoplastron; ne = neural; nu = nuchal; Pe = pectoral scute; per = peripheral; Pl = pleural scute; Ve = vertebral scute; xi = xiphiplastron

**Fig. 5:**
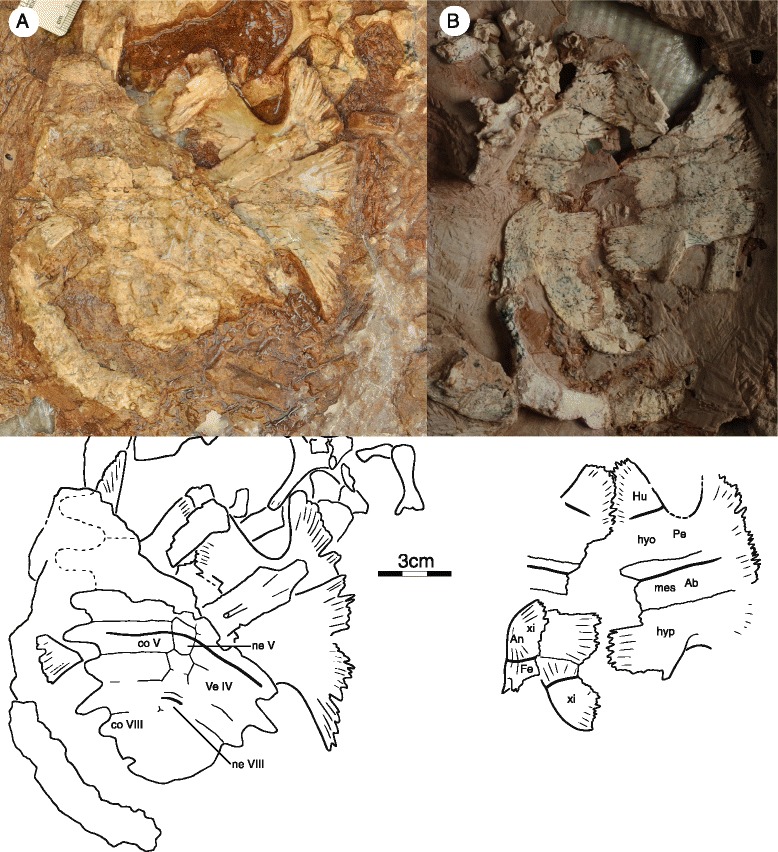
*Sichuanchelys palatodentata* n. sp., Late Jurassic (Oxfordian), Shishugou Formation, Wucaiwan, Xinjiang, China. Shell of IVPP V18095 in dorsal view (**a**) and that of IVPP V18096 in ventral view (**b**). Abbreviations: An = anal scute; co = costal; Fe = femoral scute; Hu = humeral scute; hyo = hyoplastron; hypo = hypoplastron; mes = mesoplastron; ne = neural; Pe = pectoral scute; Ve = vertebral scute; xi = xiphiplastron

All four specimens preserve at least parts of the carapace, but IVPP V18093 (Fig. 4a) and IVPP V18095 are the most informative (Fig. 5a). The original outline of the shell is somewhat unclear, as all specimens show evidence of distortion. However, despite this distortion, IVPP V18093 demonstrates that the shell had rather parallel lateral margins, that a distinct anterior shoulder was formed by peripheral II, and that a broad nuchal emargination was present. The sulci are deeply incised and delineate slightly convex scutes with week growth rings. The shell otherwise lacks any apparent shell sculpturing. In general shape, the shell resembles that of extant wood turtles, such as the extant emydid *Glyptemys insculpta* [2]. The shell bones are thin, about 1.5 mm thick in most parts of the shell, with the exception of the axillary and inguinal notches, which are about 3 mm thick. The shells of these presumed subadult individuals have carapacial, central, and posterior plastral fontanelles.

##### Nuchal

The nuchal is best preserved in IVPP V18093 (Fig. 4a). It appears to be strongly curved, but this is largely due to plastic deformation of the left side of that carapace. The nuchal is a broad, trapezoidal bone that broadly contacts peripheral I along an oblique suture anterolaterally and costal I and neural I posteriorly. The anterior margin is approximately two thirds of the posterior length of the element. The nuchal forms a broad anterior emargination together with peripherals I and II, but plastic deformation makes it appear deeper than it likely was during life.

##### Neurals

The anterior portion of the neural series is preserved in IVPP V18093 (Fig. 4a), whereas the posterior portion is preserved in IVPP V18095 (Fig. 5a). Neural I is the longest element of the series, contacts the nuchal anteriorly, neural II posteriorly, and costals I and II laterally. Neural I is intersected by the vertebral I/II sulcus. Neural II is approximately two thirds the length of neural I. A short contact of neural III with costal II prevents neural II from contacting costal III and gives neural II a rectangular outline. Neural III is only partially preserved. Given that this element has a short anterolateral contact with costal II and tapers posteriorly, we presume that it had a hexagonal outline. Furthermore, the posteriorly tapering outline of neural III indicates that neural IV had an anterolateral contact with costal III. Only the posterior portion of neural IV is preserved as the most anterior element of IVPP V18095. It lacks a posterolateral contact with costal V and therefore had a hexagonal outline. Neural V is the most posterior element to have an elongate hexagonal outline with short anterior sides. It is intersected by the vertebral III/IV sulcus. Neurals VI, VII, and VIII are greatly reduced in their anteroposterior length but are not significantly narrower than the more anterior elements. They therefore form isometric hexagons. Neural VIII has a broad posterior contact with the most anterior suprapygal element. The neural formula can be summarized as 6-4-6-6-6-6-6-6.

##### Costals

Costals I–VI are well preserved in IVPP V18093 (Fig. 4a), whereas IVPP V18095 preserves costals IV–VIII (Fig. 5a). The anterior costals were likely oriented to the anterior, but the exaggerated anterior orientation seen in IVPP V18093 is due to plastic deformation. As in most turtles, the posterior costals have a slight orientation to the posterior, as is apparent from IVPP V18095. Costal I only contacts neural I medially, whereas costal II contacts neurals I–III. All remaining costals contact two neurals medially. Costal I contacts the nuchal and peripherals I–II anteriorly and peripherals III–IV laterally. The detailed lateral contacts of the remaining costals with the peripherals are obscured by deformation, but it is apparent that the costals articulate with the peripherals via free ribs and that small costal fontanelles were retained in smaller individuals, but were closed in larger individuals. The free rib ends are better developed in IVPP V18095, a smaller specimen, indicating that the costal fontanelles were larger in juveniles.

##### Peripherals

The contacts and morphology of the anterior ten peripherals are best preserved in IVPP V18093 (Fig. 4a). The posterior peripherals are present in IVPP V18095 (Fig. 5a), but poor preservation obscures their sutures. The number of peripherals is therefore unknown. Peripheral I is wedge-shaped, but nevertheless retains a broad, posterior contact with costal I. Peripherals I–II and XIII–X are flat elements with broad dorsal exposure. By contrast, peripherals III–VII are elongate elements with only minor dorsal exposure. The ventral view of the bridge region is not sufficiently preserved in any specimen and the ventral contacts of the peripherals with the plastron are unclear. The contacts with the costals are discussed above.

##### Pygal and suprapygals

The pygal region is poorly preserved in all specimens and no significant details can be discerned.

##### Carapacial scutes

The surface of IVPP V18093 is decorated by wide and distinct carapacial scutes that allow asserting the presence of at least four vertebral scutes, four pleural scutes, and ten marginal scutes (Fig. 4a). IVPP V18095 furthermore confirms the presence of a fifth vertebral scute (Fig. 5a). The likely presence of a cervical cannot be confirmed.

The vertebral series consists of at least five elements, of which the anterior four are approximately equal in width. All vertebrals are about twice as wide as the pleurals. Vertebral I has a lenticular to octagonal shape and is therefore anteroposteriorly longer along the midline than at its lateral margins. Vertebral I has a broad anterior contact with marginal I, a short anterolateral contact with marginal II, a broad lateral contact with pleural I, and a broad posterior contact with vertebral II. An anterior contact with the cervical was likely present as well. Vertebrals II–IV are roughly hexagonal elements that contact two pleurals each laterally. Vertebral II has the outline of a butterfly that thereby partially surrounds vertebral I. Vertebrals II and IV and notably larger than vertebrals I and III.

Each pleurals contact two vertebrals medially (Fig. 4a). Pleural I is barred from contacting marginal I through a contact of vertebral I with vertebral II. It otherwise contacts marginals II–V laterally. Pleural II likely contacts marginals V–VII, pleural III contacts marginals VII–IX, and pleural IV contacts at least marginals IX–XI. The remaining contacts of the marginals with the plastral scutes are unclear.

#### Plastron

**Fig. 6:**
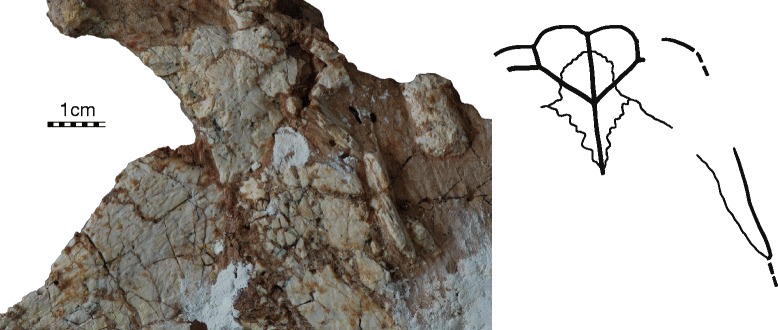
*Sichuanchelys palatodentata* n. sp., Late Jurassic (Oxfordian), Shishugou Formation, Wucaiwan, Xinjiang, China. Detail of the anterior plastral lobe of IVPP V18094. Abbreviations: ent = entoplastron; epi = epiplastron; Ex = extragular scute; Gu = gular scute. The scale is metric

The plastron of IVPP V18093 is near complete, but there is some damage to the anterior margin and the right bridge (Fig. 4b). The plastron of IVPP V18094 preserves the entoplastron best, but otherwise only consists of part of the anterior plastron lobe (Fig. 6). The plastra of V18095 and V18096 (Fig. 5) only consists of the hyo-, meso-, hypo-, and xiphiplastra. There are no meaningful visceral views of the plastron. Most plastral scutes are relatively indistinct.

##### Plastral bones

The epiplastra are relatively large elements that form the margins of the anterior half of the anterior plastral lobe (Fig. 6). The anterior, rectangular part of the epiplastra contacts the entoplastron posteromedially, and the hyoplastron posteriorly, and has a short midline contact with its counterpart. The posterior half of the epiplastron is a notably elongate, triangular posterior process that frames the anterolateral portions of the hyoplastra, similar to the process seen in *Mongolochelys efremovi*. The contacts with the hyoplastra are blunt and the epiplastra therefore easily dislocate from the anterior plastral lobe after decomposition. The anterior margin of the plastron is oriented transversely, but it is decorated by four broad lobes that correspond to the gular and extragular scutes. The margin, however, is not thickened. A distinct articular scar along the anterolateral margin of the hyoplastron in partial specimens confirms presence of a small contact between the epiplastra and hyoplastra.

The anterior portion of the entoplastron contacts the epiplastra anterolaterally but does not contribute to the anterior plastral margins (Fig. 6). The posterior portion is broadly covered by the hyoplastra in ventral view and the full extension of this element therefore remains unclear.

The remaining part of the plastron is formed by a large pair of hyoplastra, a pair of mesoplastra, a pair of hypoplastra, and a pair of xiphiplastra (Figs. 4, 5 and 6). The mesoplastra are well-developed, rectangular in shape, show no sign of narrowing medially, but do not contact one another due to the presence of a medial plastral fontanelle in all subadult specimens. The plastron is not preserved in the largest, presumable adult specimens and it therefore remains unclear if this fontanelle closes during ontogeny. The posterior plastral lobe is similar in dimensions to the anterior plastral lobe and does not exhibit an anal notch.

The sutural margins of the hyo- hypo-, and xiphiplastra are finely digitated (Figs. 4, 5 and 6). The detailed quality of the bridge articulation is unclear, but the lack of blunt sutures combined with the presence of finely fingered margins indicates that the bridge appears to have been ligamentous. The lateral margins of the plastron are too irregular or damaged to allow identifying the presence of musk duct foramina. The hyoplastron and hypoplastron form well-developed axillary and inguinal buttresses, respectively. The distal ends of the buttresses are not preserved in any specimen, but it is apparent that the anterior buttress ended anterior to peripheral IV (and therefore may have inserted in peripherals I, II, or III) and that the posterior buttresses ended posterior to peripheral VII (and therefore may have inserted in peripheral VIII to XI).

The hyoplastra meet broadly along their posterior half thereby leaving a narrow, triangular gap for the entoplastron. A clear central plastron fontanelle is formed by the hyo-, meso-, and hypoplastra that fully separates the mesoplastra along the midline, but it remains unclear if this is a juvenile feature, as the plastron is not known for any of the skeletally mature individuals. A second midline plastral fontanelle is also present between the hypo- and xiphiplastra.

##### Plastral scutes

The anterior plastral lobe of IVPP V18094 clearly reveals that a pair of gulars and extragulars are present (Fig. 6). The gulars are triangular scutes that produce clear lobes from the anterior margin of the plastron. The gulars contact the extragulars laterally, the humerals posterolaterally, and one another along the midline and cover the anterior half of the entoplastron. The extragulars are mediolaterally elongate elements that cap the anterolateral margin of the plastron. The extragulars contact the gulars medially and the humerals posteriorly, but do not contact one another medially and are restricted to the epiplastra.

The humeral/pectoral sulcus is transverse, straight, and situated midway along the hyoplastron (Figs. 4 and 5). If the remaining portion of the sulcus were to continue transversely, it would not intersect with the entoplastron. The medial portion of the pectoral/abdominal sulcus is also oriented transversely on the anterior third of the mesoplastra and enters the anterior third of the central fontanelle in IVPP V18095 and IVPP V18096. The abdominal/femoral sulcus cannot be found in any specimen, but likely was present, as in all turtles. The femoral/anal sulcus originates approximately at the anterior quarter of the xiphiplastral margin and curves anteromedially from there. The medial portion of the sulcus enters the central fontanelle and does not cross the hypoplastral/xiphiplastra suture.

Scute sulci are poorly preserved in the bridge region of all specimens and it is therefore unclear if and how many inframarginals are present. *Sichuanchelys chowi* has four pairs of inframarginals [16, 17].

#### Vertebral column

##### Cervical vertebrae and ribs

**Fig. 7:**
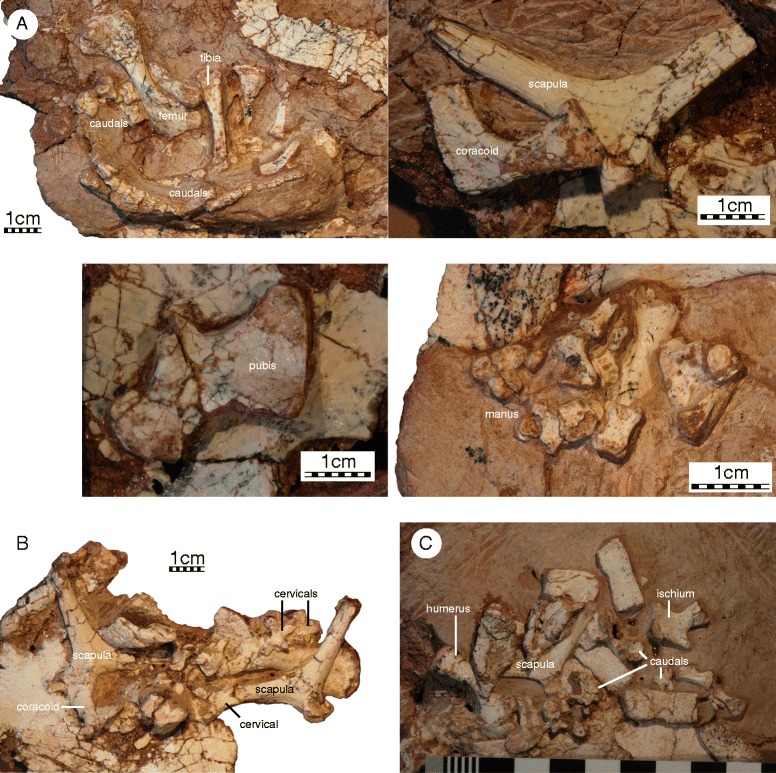
*Sichuanchelys palatodentata* n. sp., Late Jurassic (Oxfordian), Shishugou Formation, Wucaiwan, Xinjiang, China. Details from the postcranial skeleton of IVPP V18093, holotype (**a**), IVPP V18094 (**b**), and IVPP V18096 (**c**). The scale is metric

A number of disarticulated cervicals are preserved associated with specimens IVPP V18094 – V18096, but preservation is generally poor (Fig. 7b). The cervicals are typical of basal turtles in being relatively short, but high. It is unclear if cervical ribs are present, as no cervical ribs were found and the centra are too damaged to preserve parapophyses. Only a single, concave articular facet is preserved among the centra and it is therefore unclear if formed cervical articulations were present. Transverse processes are relatively long and centrally located.

##### Dorsal and sacral vertebrae and ribs

The dorsal and sacral vertebrae and ribs are either covered by sediment or too poorly preserved to allow any meaningful observations.

##### Caudal vertebrae and ribs

At least 22 caudal vertebrae are preserved with specimen IVPP V18093 (Fig. 7a, c), about 20 with IVPP V18095, and numerous isolated ones with IVPP V18096, but preservation is, once again, generally poor. Transverse processes are distinct along the anterior processes, but become increasingly smaller towards the posterior and are absent in the posterior half of the caudal column. The entire caudal column appears to have chevrons, as is evidenced by clear articular sites along the anterior half of the column and minute chevrons in articulation with the posterior caudals of IVPP V18093. The articular surfaces of only a few caudals are visible, but some appear to be amphicoelous, whereas others are slightly opisthocoelous. The great size of the basal caudals is consistent with the tail having been long (i.e., at least as long as the carapacial length).

#### Girdles and limbs

All specimens preserve remains of the shoulder girdle, but the elements are universally crushed and/or encased in sediment, making it impossible to observe all aspects (Fig. 7). It is nevertheless apparent that the scapulocoracoid is a slender triradiate complex that lacks a coracoid foramen and that the glenoid is not fused in any specimen. The scapular process is rounded distally and is only slightly longer than the acromion process. Only a minor bony lamina is developed between the dorsal process and the acromion, but it is unclear if a bony lamina or ridge runs to the glenoid, as this region is not preserved in any specimen. The distal end of the acromion is not preserved in all views and it is therefore uncertain if it is rounded distally or decorated by ridges. The scapula has a distinct neck that offsets the processes from the glenoid. The angle formed by the dorsal process and acromion is approximately 110°. The coracoid is shorter than the acromion and distally expanded to a broad fan.

Only two isolated pelvic elements are preserved, indicating that the acetabulum was not fused in subadult specimens. The isolated pelvic element associated with IVPP V18093 is interpreted as a pubis (Fig. 7a). The pubes have a broad midline contact with one another, the thyroid fenestrae are large, perhaps even confluent, and the epipubic process was not ossified. The isolated element associated with IVPP V18096 is interpreted as the ischium (Fig. 7c). The ischia have a broad midline contact and the ischial process is relatively indistinct. The posterior margin of the ischium agrees with that of *Mongolochelys efremovi* in being poorly emarginated but differs by being much smaller [32].

A number of disarticulated elements are preserved that can be attributed to the limbs, but all material is encased in sediments making it impossible to observe most details (Fig. 7). The humerus is more than twice as long as wide and has a slightly sigmoidal shaft. The medial process is flared outwards and better developed than the ventrolaterally oriented lateral process. The head is damaged in all specimens and it is therefore uncertain if a shoulder is present. The ectepicondylar canal is open, at least in the subadult specimens that preserve this bone. The ulna and radius could not be identified among the remains. A collection of bone is associated in the anterior region of IVPP V18093 that may represented a disarticulated hand, but it is not possible to identify any particular digit and the digital formula therefore remains unknown. The phalanges are nevertheless short and robust.

The only preserved femur, tibia, and fibula are too poorly preserved to allow discerning any details, beyond the observation that the femur has a slightly sigmoidal shaft (Fig. 7).

#### Presence of teeth in *Sichuanchelys palatodentata*

*Sichuanchelys palatodentata* n. sp. is striking because of the presence of palatal teeth, but the presence of such teeth is not novel among basal turtles. The Late Triassic *Proganochelys quenstedti* possesses a full set of palatal teeth that adorn the ventral surfaces of the paired vomers, palatines, and pterygoids [37]. Palatal teeth are otherwise known from the Permian proto turtle *Eunotosaurus africanus* [38] and the Middle Triassic proto turtle *Odontochelys semitestacea* [39]. This is the basal amniotic condition [40]. The skull of all other known Triassic turtles is either missing or too poorly preserved to allow rigorously assessing the presence of palatal teeth. The gradual loss of teeth was previously documented only by the Early Jurassic *Kayentachelys aprix*, which clearly lacks vomerine and palatine teeth, but retain a reduced count of pterygoid teeth. All more derived and younger turtles were thought to lack palatal teeth [22]. The presence of pterygoid teeth in *S. palatodentata* extends the plesiomorphic retention of these structures in at least one lineage to the Late Jurassic, but we do not believe that this has any particular functional significance.

### Phylogenetic analysis

**Fig. 8:**
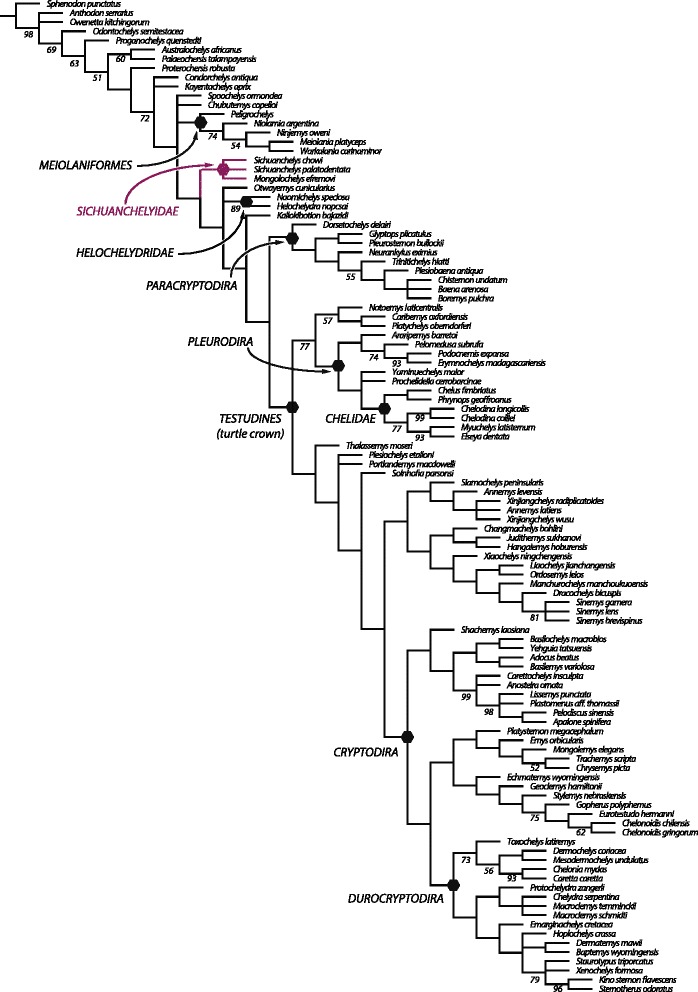
A strict consensus tree of 550 most parsimonious trees with 960 steps resulting from phylogenetic analysis. 5 wildcard taxa were pruned from the consensus

Our parsimony analysis (see Methods below) resulted in 550 most parsimonious trees with 960 steps. *Heckerochelys romani*, *Eileanchelys waldmani*, *Indochelys spatulata*, *Patagoniaemys gasparinae*, and *Xinjiangchelys junggarensis* act as wild-card taxa and were therefore pruned from the consensus tree (Fig. 8). *Sichuanchelys palatodentata* was retrieved in a polytomy with *Sichuanchelys chowi* and *Mongolochelys efremovi* within the clade Sichuanchelyidae along the stem lineage of turtles. Bootstrap resampling reveals that support for this group is strong (73 %) if *S. chowi* is removed from the analysis, likely because this species is incompletely known. Helochelydridae and *Kallokibotion bajazidi* are placed in successively more crownward positions relative to Sichuanchelyidae. Turtles with the paracryptodiran carotid circulation are retrieved as monophyletic as the immediate sister of crown Testudines. Meiolaniformes is here restricted to Meiolaniidae and *Peligrochelys walshae* and are placed in a more basal position than Sichuanchelyidae. *Spoochelys ormondea* and *Chubutemys copelloi* form a polytomy with Meiolaniformes and the clade consisting of all other more derived taxa. Morphological support for the placement of *M. efremovi* into a clade with *Sichuanchelys palatodentata* is high and we are therefore confident in that *M. efremovi* is not nested within Meiolaniformes as previously proposed (e.g., [26, 41–43]).

### Biogeographic analysis

**Fig. 9:**
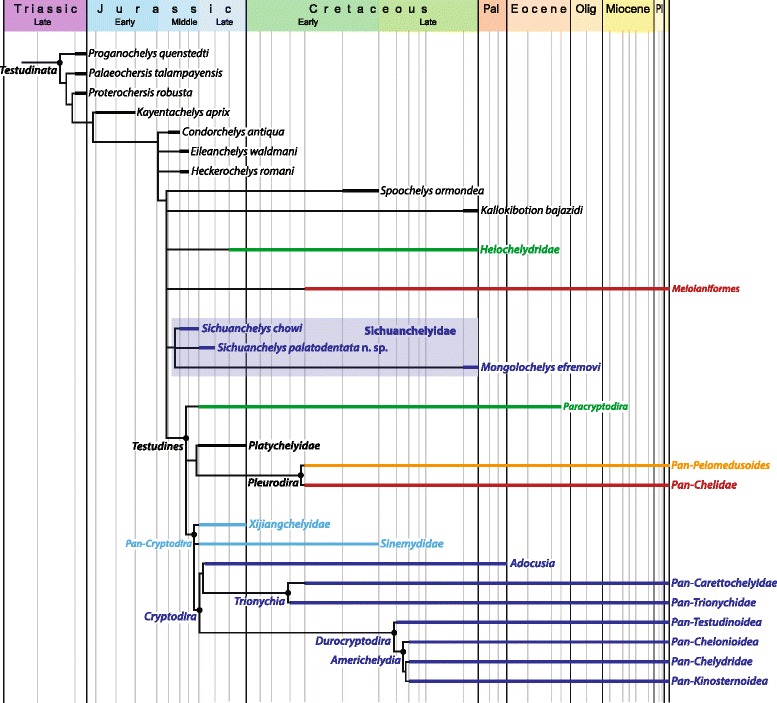
A composite phylogenetic consensus of turtle relationships highlighting the most important clades discussed in the text and their stratigraphic distribution as derived from the inclusion of fragmentary material. To aid understanding the text, internal relationships and nodes are only provided within Pan-Cryptodira. Support for all clades or polytomies is provided in the text (see Biogeographic analysis)

The fossil record of turtles is relatively good and multiple attempts have therefore been made to discern global [6, 13] or regional biogeographic patterns [26, 31, 44–46]. However, new fossils, insights into the paleoecology of fossil turtles, and novel phylogenetic hypotheses allow us to synthesize a global biogeographic model that reveals that the diversification of turtles was primarily driven by vicariance caused by the breakup of Pangaea. We demonstrate below that this pattern is apparent at two successive phylogenetic levels. Given that some parts of the turtle tree remain controversial, in particular the inclusiveness of Pan-Cryptodira and the interrelationships of sichuanchelyids, helochelydrids, and meiolaniforms [6–12, 25, 26, 41–43, 47], we attempt to present a model that is relatively immune to future changes in the understanding of phylogenetic patterns by highlighting the distinct evolutionary history of seven clades of turtles. These conflicting signals are reflected in the composite topology we utilize herein (Fig. 9), which combines the result of previous studies with our current strict consensus tree (see Phylogenetic analysis above). The monophyly of each clade is discussed below and phylogenetic ambiguities are highlighted.

The taxonomic identity of the fossils utilized herein is not controversial, as we only employ specimens that exhibit clear, apomorphic characters. The vast majority of informative fossils is fragmentary, however, and has therefore not yet been integrated into global phylogenetic analyses. We therefore refrain for the moment from providing a probabilistic model of historical biogeography and rather present a narrative account based on all available data.

There has been full consensus over the course of the last 100 years that extant turtles can be grouped into two clades, Pleurodira and Cryptodira, but the vast majority of fossil taxa were traditionally shoehorned into this dichotomy and turtles were therefore thought to lack a substantial stem lineage. All species-level phylogenies of the last decade [6–12, 25, 26, 41–43, 47] have converged upon the novel conclusion that the stem lineage leading to the crown is populated by a diverse assemblage of fossil turtles that inhabited all continents from the Triassic to the Pleistocene. All conflicting hypotheses [4–7] have been shown to converge upon this result through minor modifications, in particular the addition of characters, taxa, or new specimens [48, 49]. Although a certain amount of ecological plasticity is apparent, the basal stem turtle lineage is dominated by terrestrial forms, whereas crown turtles and their immediate sister groups are dominated by freshwater aquatic forms [50]. We herein discuss the parallel diversification of derived, aquatic turtles and basal, terrestrial turtles separately for convenience and highlight important developments that occurred in parallel.

It is important to emphasize that the strong biogeographic signal we discuss herein only emerges once all littoral to marine clades are omitted from consideration, as these obscure the continental pattern that otherwise emerges based on freshwater aquatic and terrestrial forms alone. The littoral to marine groups we identify are listed further below under dispersal.

### The biogeography of derived, freshwater aquatic turtles

The vast majority of recent molecular and morphological studies (see [14, 47] for most recent summary) support the monophyly of the primary clades that make up crown Testudines: Pan-Cryptodira and Pan-Pleurodira, which in turn is comprised of Pan-Chelidae and Pan-Pelomedusoides. The fossil record furthermore reveals the presence of another clade that diverged near the base of the crown group: Paracryptodira (e.g. [8, 10, 42]). Our review of the fossil record indicates that these four clades can be traced back to four distinct biogeographic areas in the Late Jurassic to Early Cretaceous. The monophyly of each group and their biographic distribution is discussed below.

#### Pan-Chelidae

**Fig. 10:**
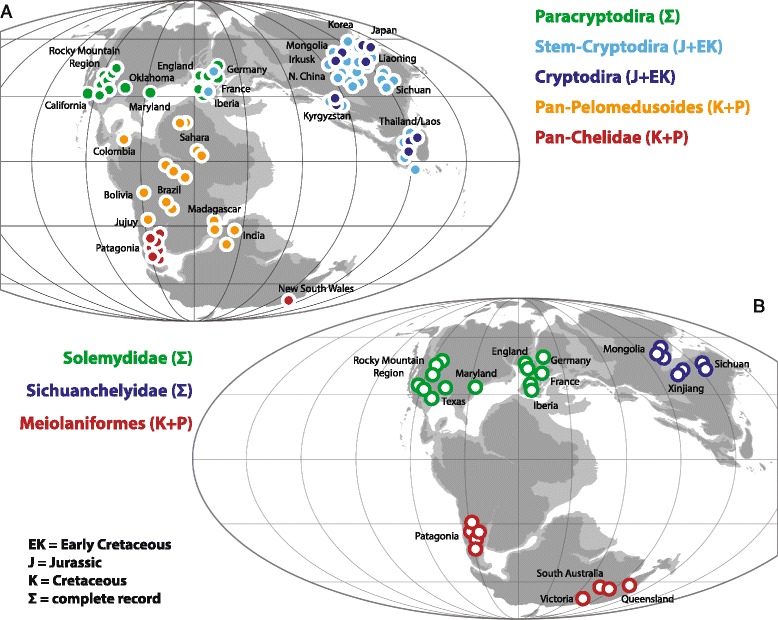
The vicariant origin of the primary clades of turtles. **a** the early or complete fossil record of the clades Pan-Chelidae, Pan-Pelomedusoides, Paracryptodira, Pan-Cryptodira and Cryptodira imposed upon a paleogeographic reconstruction for the Late Jurassic (modified from [142]) highlighting the biogeographic areas of southern Gondwana, northern Gondwana, Euramerica and Asia, respectively. **b** the complete fossil record of the clades Meiolaniformes, Helochelydridae, and Sichuanchelyidae imposed upon the paleogeographic reconstruction for the Late Jurassic (modified from [142]) illustrating the biogeographic areas of southern Gondwana, Euramerica and Asia, respectively

**Fig. 11:**
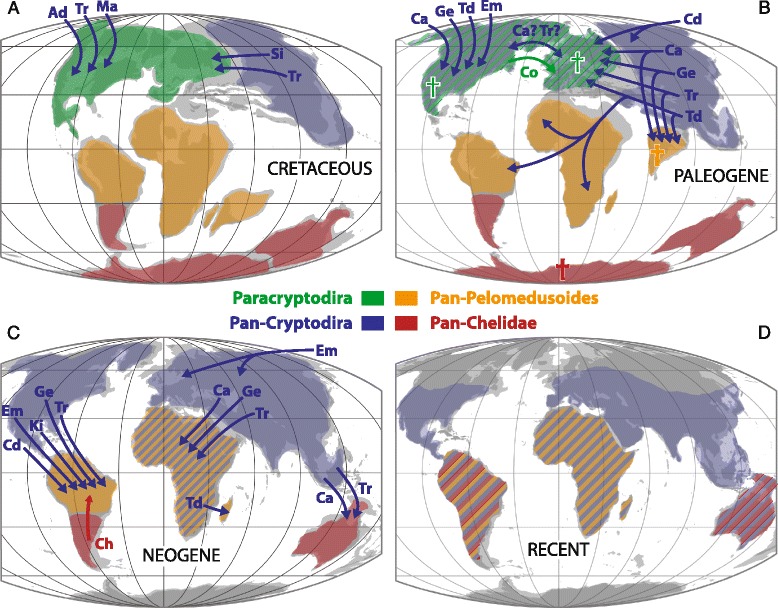
The biogeographic history of derived turtles following their primary origin through dispersal. **a** dispersal during the Cretaceous; **b** dispersal during the Paleogene; **c** dispersal during the Neogene; **d** the current distribution of turtle clades. *Shaded* areas highlight the distribution of turtle clades at the beginning of a particular time bin as inferred from the fossil record (see Fig. 10). *Arrows* highlight paths of freshwater aquatic or terrestrial dispersal. For simplicity, all island taxa and groups adapted to coastal and marine settings are disregarded. Abbreviations: Ad = Adocusia; Ca = (Pan)-Carettochelyidae; Cd = (Pan)-Chelydridae; Ch = Chelidae; Co = *Compsemys*; Em = Emydidae; Ge = (Pan)-Geoemydidae; Ki = Kinosternidae; Ma = Macrobaenidae; Si = Sinemydidae; Td = (Pan)-Testudinidae; Tr = (Pan)-Trionychidae

The monophyly of crown Chelidae has never been controversial. Chelids are freshwater aquatic turtles that today occur throughout South America and Australasia [2] (Fig. 11d), but its total group, Pan-Chelidae, was restricted, without exception, to Australasia and the southern half of South America for most of its history (Fig. 10a) and only invaded the northern half of South America during the Neogene [44]. The oldest known fossils referable to the chelid lineage are from the Aptian/Albian of Argentina [51, 52] and the Albian of Australia [53] and the group therefore lacks an apparent center of origin (contra [13, 54]). The known distribution of pan-chelids predicts the former presence and extinction of the group on Antarctica, as a transoceanic dispersal event is highly unlikely between South America and Australia. The original distribution of pan-chelids was therefore restricted to southern South America, Australia, and presumably Antarctica, a landmass previously termed “Southern Gondwana” [44]. The early history of the group is consistent with a vicariant origin of South American versus Australian chelids in the Early Cretaceous, as predicted by molecular phylogenies [55, 56] and molecular calibration studies [57], but contrast with morphological data [58]. Rigorous phylogenetic analysis of all Cretaceous representatives is needed to further test this hypothesis.

#### Pan-Pelomedusoides

In contrast to chelids, pelomedusoids today occur throughout Africa, Madagascar, and the northern half of South America [2] (Fig. 11d). Although various littoral to marine representatives helped this group of turtles to achieve a near-global distribution during much of the Cretaceous and Tertiary [59, 60] through a dizzying array of marine dispersal events (see below), the freshwater aquatic representatives of this clade were consistently restricted to northern South America, Africa, Madagascar, and India throughout their evolutionary history [59, 60] (Fig. 10a), a landmass named “Northern Gondwana” [44]. Throughout the mid-Cretaceous numerous stem-pelomedusoid “species pairs” are apparent between northern South America and Africa that highlight the faunal ties between these continents (e.g., Araripemydidae, Cearachelyini, and Euraxemydidae [59, 61]), but focused analysis will be necessary to infer vicariance as the direct cause of speciation within these clades (see also [62]). There currently is no evidence that the primary split of Pelomedusoides into the pelomedusid and podocnemidid lineages was caused by vicariance, as the fossil record of freshwater aquatic pan-podocnemidids is broadly distributed across Northern Gondwana [60]. Unambiguous pan-pelomedusids, by contrast, are only known from the Neogene of Africa [57].

There has been much debate whether the unusual distribution of extant podocnemidids in South America and Madagascar [2] is, among others, the result of vicariance [63], differential extinction within a formerly widespread group [64, 65], dispersal from Africa to Madagascar [66], or a mixture of vicariance and dispersal across Antarctica [67]. One reason why this conundrum remains unresolved is because there is no agreement as to the phylogenetic relationships among the three primary lineages of extant podocnemidids (i.e., *Erymnochelys madagascariensis*, *Peltocephalus dumerilianus*, and *Podocnemis* spp.) and because competing topologies imply different biogeographic histories. This problem is only further compounded by the lack of fossil forms that unambiguously represent the stem lineages of the Malagasy *Erymnochelys madagascariensis* and the South American *Peltocephalus dumerilianus*, which may perhaps reach back into the Cretaceous*.* Until the fossil record provides a more definitive answer, we here refrain from supporting any particular biogeographic scenario. However, we feel that the complete lack of fossil podocnemidid turtles in Southern Gondwana [60, 68] make dispersal from South America to Madagascar via Southern Gondwana highly unlikely.

The primary distribution of pan-pelomedusoids in Northern Gondwana and pan-chelids in Southern Gondwana is best interpreted as the result of a vicariance event, as previously proposed [44], and this event must have occurred prior to the Barremian [69]. A previous study [69] speculated that vicariance was driven by a volcanic event that is documented by large volcanic fields in southern Brazil, but we do not think this to be likely, as this volcanic event only lasted about one million years [70]. As an alternative, we speculate that the subtropical desert zone that crossed the southern portion of Gondwana during much of the late Mesozoic [71–73] persistently divided the freshwater habitats of early pleurodires into a larger northern and a smaller southern range. This desert zone apparently influenced the biogeographic distribution of other groups of organisms, including dinosaurs [74].

#### Paracryptodira

Although the phylogenetic relationships of Paracryptodira relative to Pleurodira and Cryptodira remains unresolved, there is broad agreement that the group is monophyletic, not situated within crown Cryptodira or crown Pleurodira, and that their freshwater aquatic habitat preferences are a derived character shared with crown turtles [7, 8, 10, 11, 25, 26, 42, 47, 75]. This clade is comprised of a diverse assemblage of medium-sized turtles that were restricted, without exception, to Euramerica (Europe + North America) throughout their evolutionary history (Fig. 10a) and that provide evidence for the close biogeographic relationships of these land masses, as previously noted [6]. The oldest known paracryptodires are known from Upper Jurassic deposits in both North America and Europe [31, 46, 76], but isolated finds extend the range to the Middle Jurassic of Europe [77]. This biogeographic area was fully separated from Asia by the Turgai Strait and from Gondwana by the Central Atlantic and the Tethys for much of the Late Jurassic and Early Cretaceous [78]. Paracryptodires were particularly diverse throughout the Late Cretaceous and Paleogene [76, 79–81], but the group went extinct prior to the Oligocene [82]. The paracryptodiran clade Baenidae is restricted to the Early Cretaceous to Paleogene of western North America (Laramidia), but there is no reason to interpret this as evidence for vicariance (contra [6]), as Baenidae lacks a sister group on a nearby landmass. The currently accepted sister of Baenidae, Pleurosternidae, instead shows a broad distribution across Euramerica.

#### Pan-Cryptodira

The composition of the total group of Cryptodira, i.e., Pan-Cryptodira, is currently one of the most controversial subjects in turtle phylogeny. Although the broad sample of basal, terrestrial forms discussed below has been removed from the cryptodiran stem group with confidence (see above), there is still much uncertainty regarding a similarly broad sample of freshwater aquatic forms [7, 8, 10, 11, 25, 26, 42, 47, 75], in particular xinjiangchelyids, sinemydids, and macrobaenids (sensu [11]). The character evidence that places xinjiangchelyids and sinemydids along the phylogenetic stem of Cryptodira is quite convincing, because these taxa document the step-wise acquisition of cryptodiran characters throughout the Middle to Late Jurassic. The character evidence is particularly strong in the basicranial region and these turtles have therefore been collectively united with crown cryptodires in the clade Eucryptodira [83]. Yet, pleurodires have been routinely recovered deep within Eucryptodira (e.g., as sister to Testudinoidea) (e.g., [8, 11, 25, 47]), although the latest study demonstrated that this signal is an analytical artifact [12]. The extremely rich Middle Jurassic to Late Jurassic fossil record of freshwater aquatic pan-cryptodires is restricted to Asia [29, 84, 85], with the exception of marine plesiochelyids and eurysternids found in the Late Jurassic of Europe [31, 46] (Fig. 10a). It was not before the Early Cretaceous that isolated freshwater eucryptodire taxa dispersed into Europe [86].

The early record of unambiguous crown cryptodires is fully restricted to Asia until the Early Cretaceous [84–88] (Fig. 10a), but this pattern is soon after obscured by the successful dispersal of marine, freshwater, and terrestrial pan-cryptodires throughout the Late Cretaceous to Pleistocene (see section below; Fig. 11a–c). The currently available fossil record does not support the hypothesis that the basal split within crown Cryptodira (i.e., the split between Pan-Trionychia and Pan-Durocryptodira) was caused by vicariance. It is notable, however, that the primary clades of Durocryptodira (i.e., Pan-Testudinoidea and Pan-Americhelydia), show an asymmetric distribution, with the early record of the former being restricted to Asia [6] and that of the latter to North America [57]. It will only be possible to establish whether this distribution is due to vicariance (as suggested for Testudinoidea [6]) or dispersal (as previously suggested [13]) through the discovery of unambiguous stem-americhelydian turtles. The early presence of such taxa in Asia positively would imply dispersal of the americhelydian ancestor to North America, whereas their absence in Asia would corroborate vicariance. A previous analysis [6] suggested that Chelydridae, one of the primary clades of Americhelydia, originated through vicariance, but given that most Late Cretaceous chelydrid localities also contain fossils of the sister group Kinosternoidea (e.g., [89–91]), it is apparent that these two clades originated sympatrically, at least at a continental scale, and lack a vicariant distributional pattern.

### Vicariance-driven primary divergence of derived, freshwater aquatic turtles

The exclusive presence of fresh-water aquatic pleurodires in Gondwana, paracryptodires in Euramerica during the late Middle Jurassic to Early Cretaceous, and the restriction of pan-cryptodires to Asia during the Middle to Late Jurassic (Fig. 10a), combined with the difficulty of phylogenies to rigorously resolve the phylogenetic relationships of these three taxa, is strong evidence for the nearly coeval split of these lineages due to vicariance around the Middle Jurassic. Based on the distribution of fossils (Fig. 10a) and the estimated timing of the primary divergence of crown-turtles, the Early to Middle Jurassic separation of Gondwana and Laurasia was therefore the driving factor for the split of crown turtles into pan-pleurodires and pan-cryptodires [14, 57]. The freshwater aquatic habitat preferences of the common ancestor of this clade of turtles [50] either suggest barriers created by salt water or terrestrial deserts. The marine barriers that appear to have driven these vicariance events are the opening of the North Atlantic, which originated from the initial breakup of Pangaea into Gondwana and Laurasia, and the establishment of the Turgai Strait, which split Asia from the remaining northern continents during the Jurassic and Cretaceous. Independent geological evidence places the origin of these barriers at the Middle Jurassic [78, 92]. The subsequent split of pleurodires, by contrast, appears to have been driven by a terrestrial barrier (see above), which likely originated after the Middle Jurassic, but prior to the late Early Cretaceous. This leads us to the novel conclusion that the primary divergence of derived turtles into four clades (i.e., Paracryptodira, Pan-Cryptodira, Pan-Pelomedusoides, and Pan-Chelidae) was driven by vicariance alone in the form of continental breakup and the formation of intra-continental barriers. There currently is no evidence for further vicariance within these clades, although we note possible examples above. The extensive destruction of the vicariant signal through secondary dispersal events is discussed below.

### The biogeography of basal, terrestrial turtles

One of the more surprising conclusions of the last 10 years of phylogenetic research is that turtles have a substantial stem lineage [7, 8, 10, 11, 25, 26, 42, 47, 75]. This stem lineage includes the expected sequence of basal forms that help span the morphological gap between the most turtle-like proto turtle, the Middle Triassic *Odontochelys semitestacea* [39], and the turtle crown, such as the Late Triassic *Proterochersis robusta* Fraas, 1913 [93], *Proganochelys quenstedti*, and *Palaeochersis talampayensis* Rougier et al., 1995 [94], the Early Jurassic *Kayentachelys aprix*, and the Middle Jurassic *Heckerochelys romani* Sukhanov, 2006 [95], *Condorchelys antiqua* Sterli, 2008 [9], and *Eileanchelys waldmani* Anquetin et al., 2009 [96]. In addition, the stem lineage includes a number of lineages that diversified throughout the Mesozoic and Cenozoic, in parallel with crown turtles, and of which the last representative died out as recently as the late Pleistocene [5, 97].

We herein recognize three post-Jurassic lineages that can be traced back to geographic areas that coincide with those already established above for more derived aquatic turtles: Meiolaniformes, Helochelydridae, and Sichuanchelyidae (for phylogenetic definitions of all three clades see above). In current phylogenies, these three lineages typically are retrieved as a clade [8–11, 26, 42], but also occasionally as a paraphyletic grade [6], or even a polyphyletic assemblage [7]. The analysis we present herein, the most comprehensive to date in regards to this taxa, retrieves a paraphyletic arrangement (see above), but we remain cautious as the phylogeny of basal turtles is in a state of flux. To a certain degree, this phylogenetic ambiguity is not problematic, because the monophyly of each of these three lineages appears to be unambiguous and because these three lineages can be inferred to have been present by the Middle Jurassic. At ‘best’ (i.e., if monophyletic) these lineages therefore document true vicariance among a stem turtle lineage. At ‘worst’ (i.e., if paraphyletic or polyphyletic) they establish a vicariance-like pattern among stem turtles. To remain conservative for the moment, we consider these three clades to have unresolved relationships with respect to each other outside of crown Testudines (Fig. 9). To highlight that additional turtles exist with disputed phylogenetic relationships and at the same evolutionary level, we also include the Cretaceous turtles *Kallokibotion bajazidi* and *Spoochelys ormondea* into this polytomy (Fig. 9). Further analyses may reveal these turtles to be attributable to Meiolaniformes, Helochelydridae, and Sichuanchelyidae or to represent additional lineages of basal turtles that survived long past the origin of crown turtles.

#### Meiolaniformes

The clade Meiolaniformes was historically known from its Cenozoic representatives only, the “cow-horned turtles”, which are known exclusively from Australasia and the southern half of South America [5] (Fig. 10b). A number of recent finds have been important for the understanding of this clade, as they bridge the morphology chasm that exists between classic meiolaniid turtles and the turtle stem lineage. The most important taxon is *Chubutemys copelloi* [7], which is based on a partial skeleton from the Early Cretaceous (Aptian) of Argentina (also see [43]). A series of additional taxa based on more fragmentary material may further document the persistent presence of the lineage in this biogeographic area, including *Patagoniaemys gasparinae* Sterli and de la Fuente, 2011 [41] and *Trapalcochelys sulcata* Sterli et al., 2013b [98] from the Late Cretaceous (Campanian–Maastrichtian) of Argentina, and *Peligrochelys walshae* Sterli and de la Fuente (2013) [26] from the Paleocene of Argentina. The Early Cretaceous Australian taxon *Otwayemys cunicularius* Gaffney et al., 1998 [99] is identified in our analysis as problematic, but is associated with Meiolaniformes in other analyses [7, 10, 26]. The complete biogeographic distribution of this clade of turtles fully coincides with the core area of chelid turtles and also predicts the presence of the group on Antarctica at some time, as previously already noted [26, 43]. The realization that this clade of turtles is restricted to South America and Australia is a novel result obtained through the addition of *Sichuanchelys palatodentata* to the analysis, as the addition of this taxon pulls the Asian *Mongolochelys efremovi* and the European *Kallokibotion bajazidi* out of Meiolaniformes.

#### Helochelydridae

Helochelydrid turtles have been known from the Cretaceous of Europe since the 1850s and from North America since the 1900’s [100]. Although the presence of this transcontinental grouping had already been proposed during the first half of the 20th century [33], it was not well accepted until recently, though under the name Solemydidae [6, 28, 34]. The best-known representatives of this clade are *Naomichelys speciosa* Hay, 1908 [76] which is known from a complete skeleton from the Early Cretaceous (Aptian–Aptian) of Texas [101] and *Helochelydra nopcsai* Lapparent de Broin and Murelaga, 1996 [28], known from a partial skeleton [33] and a well-preserved skull from the Barremian of England [100]. The highly distinct surface sculpture of helochelydrid shells allows confident referral of fragmentary remains and the current record of the group spans from the Late Jurassic (Tithonian) to the Late Cretaceous (Maastrichtian) of Euramerica [100, 101]. The geographic distribution of Helochelydridae and Paracryptodira fully overlap with one another over their entire known history (Fig. 10).

#### Sichuanchelyidae

*Mongolochelys efremovi* from the Late Cretaceous (Maastrichtian) of Mongolia had previously been a paleobiogeographic enigma, because it was the only stem turtle known from the Cretaceous of Asia and therefore biogeographically isolated from all potential sister groups (Fig. 10b). This taxon has repeatedly been discussed as a primitive relict within the Asian turtle fauna [26, 31, 43, 102], but the absence of fossils to the contrary made it impossible to exclude that this pattern was due to dispersal, a possibility made plausible by the late occurrence and highly nested position of *M. efremovi* within a clade otherwise dominated by southern taxa.

Our conclusion that the Late Jurassic *Sichuanchelys palatodentata* and, by extension, the Middle Jurassic *Sichuanchelys chowi* are sister to the Late Cretaceous *Mongolochelys efremovi* is highly significant. Even though *S. palatodentata* and *M. efremovi* are united by a number of unique apomorphies (e.g., enlarged squamosals that form a collar, near contact of jugal and quadrate, enlarged antrum postoticum that is not enclosed laterally, entry of palatine artery through ventrally open foramina caroticum laterale, paired pits on the ventral side of the basisphenoid, nuchal notch delimited by peripherals II, and, potentially, retention of plastral fontanelles), the highly primitive morphology of *S. palatodentata*, including the conspicuous retention of pterygoid teeth, anchors the clade at a more basal position along the turtle tree, not deeply nested within Meiolaniformes. Although a basal position had previously been inferred for *S. chowi* from shell morphology [16, 103], its strongly supported relationship with *M. efremovi* is highly surprising and extends the ancestral lineage of this taxon by ca. 100 million years into the Middle Jurassic. *Mongolochelys efremovi* is therefore no longer an out of place turtle and further fossil discoveries in Asia will likely further fill the record of Sichuanchelyidae. While the herein proposed phylogenetic relationships of Sichuanchelyidae relative to Helochelydridae, Meiolaniformes, and crown Testudines may change through the addition of further data in the future, the unambiguous presence of sichuanchelyids in the Middle Jurassic of Asia [16] and of helochelydrids in the Late Jurassic of Europe [100] highlights that the three groups of basal turtles discussed herein (i.e. Meiolaniformes, Sichuanchelyidae, and Helochelydridae) had split from one another no later than the Middle Jurassic.

### Biogeographic parallels between basal and derived turtles

Previous authors have already noted the overlapping distribution of Helochelydridae with Paracryptodira [6] and of Meiolaniformes with Pan-Chelidae [26] and we here are able to highlight an equivalent overlapping distribution between the newly established clade Sichuanchelyidae and Pan-Cryptodira. Interestingly, the two clades of basal turtles from the northern hemisphere not only correspond with their crown-ward counterparts in their geographic distribution, but also in their temporal distribution, by originating in the Middle to Late Jurassic. The Jurassic terrestrial record of Gondwana is still too poor to allow tracing groups of turtles with confidence on this landmass. Although it remains unclear if the three groups of basal turtles discussed herein form a clade, the close temporal and spatial association with derived aquatic turtles makes it likely that these groups were separated from one another by the same processes that vicariantly separated Pan-Pleurodira, Pan-Cryptodira, and Paracryptodira (i.e., the formation of the Turgai Strait and the opening of the Atlantic). However, whereas it would be possible to postulate vicariance as the cause for the breakup of a monophyletic group of basal turtles, a complicated pattern of regional extinctions would have to be postulated if these turtles form a paraphyletic grade. Regardless of the outcome of this debate, the non-overlapping distribution of these three clades makes it apparent that each diversified following the Middle Jurassic from a different ancestor stranded on a different part of the globe.

It is notable that Pan-Pelomedusoides is the only clade of derived aquatic turtles that lacks a basal counterpart. If the phylogeny of basal turtles was driven by the same processes as crown turtles, our model would predict the presence of a hereto undiscovered clade of terrestrial, basal turtles that originated in Northern Gondwana no later than the Early Cretaceous and that went extinct at some time prior to the Recent.

### The destruction of the vicariance signal through dispersal

The three clades of basal turtles outlined herein (i.e., Meiolaniformes, Helochelydridae, and Sichuanchelyidae) were restricted throughout their evolutionary history to the land areas upon which they originated through vicariance (Figs. 9 and 10). Some amount of internal movement can only be posited with confidence for Meiolaniidae, as the discovery of highly derived meiolaniids on islands off the coast of continental Australia is best explained by dispersal [5].

The fossil record of pan-chelid turtles is restricted to the southern portions of South America prior to the Neogene (Fig. 10a). Molecular phylogenies retrieve monophyletic clades of South American and Australian chelids [55, 56] and therefore imply the vicariant origin of modern chelids. By contrast, competing morphological hypotheses [58] either demand the diversification of chelids prior to the breakup of Southern Gondwana with select extinction on both continents, or diversification after the breakup of Southern Gondwana with multiple subsequent dispersal events, neither of which is currently supported by the fossil record. Multiple lineages of chelid turtles successfully invaded the Amazon Basin during the Neogene [44]. Our model predicts that chelids must have been present on Antarctica during the Early Cretaceous, but have since gone extinct (Fig. 11).

The representatives of two clades of pan-pelomedusoid turtles, Bothremydinae and Stereogenyina, are regularly retrieved from littoral to marine sediments [59, 60, 104, 105] and we therefore reconstruct these clades as being ancestrally adapted to near-shore conditions, even if derived representatives within these clades are occasionally found in freshwater deposits [59]. If these two clades are disregarded from consideration, the entire pan-pelomedusoid fossil record is restricted to Northern Gondwana (i.e., northern Southern America, Africa, Madagascar, and India), which contrasts with the southern distribution of pan-chelids already outlined above [38] (Fig. 10a). A number of marine dispersal events can be reconstructed between Africa, Europe, India, Madagascar, and North and South America within Bothremydinae [104, 105] and between Africa, India, Puerto Rico, and South and North America within Stereogenyina [106]. The question whether the current distribution of podocnemidids in South America and Madagascar is due to vicariance or dispersal remains unresolved (see discussion above).

The entire fossil record of paracryptodires is restricted to the original land area of the clade Fig. 10a). Although initially connected, North America and Europe were slowly fragmented during the Jurassic and Cretaceous by the opening of the North Atlantic Ocean and the Labrador Sea and by the incursion of epicontinental seaways that criss-crossed Euramerica. As currently preserved, the paracryptodiran record contracted from its maximum in the Late Jurassic (i.e., all of Europe and North America) to a minimum in the Late Cretaceous and Paleogene (i.e., only the western portion of North America or Laramidia) [31, 46, 76, 77]. The isolated presence of *Berruchelus russelli* Pérez-García, 2012 [81] in the Paleocene of France, combined with its sister group relationship with the highly derived North American *Compsemys victa* [24], is therefore best explained through a dispersal event from North America to Europe during the early Paleocene [81] (Fig. 11b).

In contrast to all other clades of turtles, pan-cryptodires dispersed prolifically throughout their evolutionary history and have come to dominate turtle faunas on most continents [2] (Fig. 11). The Turgai Strait to the west initially defined the primary land area of pan-cryptodires and only the marine Eurysternidae/Plesiochelyidae/Thalassemydidae overcame this barrier prior to the Early Cretaceous by dispersing to Europe [31, 46]. By the Early Cretaceous the Turgai Strait became leaky as demonstrated by an eclectic assemblage of unrelated fresh water taxa in the European fossil record [86, 107]. A poorly known littoral/marine clade of pan-cryptodires (Angolachelonia or Sandownidae) is known from the Cretaceous to Paleogene of Europe, North America, South America, and Africa [108–110], whereas the fully marine turtles of the clade Protostegidae are known from the Cretaceous of South America, North America, Europe, North Africa, Australia and Japan [111–114]. Unambiguous representative of the extant marine turtle clades Pan-*Dermochelys* and/or Pan-Cheloniidae have been reported from all continents during the Cenozoic [115, 116].

Among freshwater aquatic and terrestrial lineages, trionychids, adocids (*Adocus*), nanhsiungchelyids (*Basilemys*), and macrobaenids successfully invaded North America from Asia via the Bering Land Bridge during the Cretaceous [45], and carettochelyids (*Anosteira*), emydids, geoemydids, and testudinids followed during warm periods of the Paleogene [14, 117] (Fig. 11b, c). The unidentified ancestral lineage of Americhelydia must have followed this path as well. Given that western North America was separated from eastern North America by the intercontinental seaway, it is possible that various taxa migrated to eastern North America via the Arctic [118]. The Bering Land Bridge was utilized by chelydrids and the *Emys orbicularis*-lineage to disperse to Eurasia during the Paleogene [119, 120] and Neogene [121], respectively (Fig. 11c).

Chelydrids (*Chelydra*), emydids (*Trachemys*), geoemydids (*Rhinoclemmys*), and kinosternids (*Kinosternon*) successfully colonized South America from North America during the Great American Interchange [68] (Fig. 11c). Isolated remains of trionychids in South America document a failed attempt to follow this path as well [122]. Interestingly, no cryptodires are known to have dispersed from South America to North America during the entire Cenozoic (e.g. [123]). The northernmost distribution that South American testudinids (*Chelonoidis*) achieved was into the Caribbean [124] (Fig. 11c).

Carettochelyids (*Allaeochelys*), geoemydids, testudinids, and trionychids invaded Europe [31, 46] and India [87, 125, 126] from Asia during the Eocene. Although it is not clear if they dispersed from Asia or from Europe, testudinids arrived in Africa during the Eocene [127] and carettochelyids, geoemydids, and trionychids followed in the Miocene [66] (Fig. 11c). The dispersal of testudinids from Africa to South America is currently dated at the Oligocene [68] whereas their arrival on Madagascar is calculated to have occurred in the Neogene based on molecular data [128] Fig. 11c). In more recent history, testudinids have successfully colonized Bermuda, the West Indies, the Galápagos and Mascarene Islands, and the Seychelles (see [129] for summary), although good reasons exist to speculate that humans additionally meddled with the natural distribution [130]. For simplicity, we omitted all dispersal events to small islands in our summary (Fig. 11c). Trionychids and carettochelyids finally migrated to Australia/New Guinea no later than the Eocene [131] and Miocene [132], respectively (Fig. 11c).

## Discussion

Our study differs from previous biogeographic analyses by revealing that the early diversification of turtles from the Middle Jurassic to Early Cretaceous was driven by vicariance, but that this pattern is secondarily obliterated through extensive dispersal throughout the Late Cretaceous and Tertiary. Although many important aspects of this pattern had previously been recognized, in particular in regards to post-Jurassic dispersal [6, 13, 31, 45, 46, 82, 117, 119], little vicariance had previously been proposed [14, 44]. We identify three factors that helped us discover the primary vicariance pattern.

### New insights into the fossil record

Fossil turtles comprise a significant portion of the fossil vertebrate fauna globally, but little attention had been accorded to the group throughout the 20th century. Much therefore remains to be learned about the fossil record of many groups of turtles [57]. A renewed interest in the group over the course of the last 30 years resulted in the description of new material and the re-evaluation of existing collections. Affordable international travel and photography furthermore have made it easier to compare directly material from different regions. These aspects have had a significant impact upon our study, as many groups of turtles and transcontinental relationships were only recognized in the last decades.

### A focus on the terrestrial signal

In contrast to all previous studies, we are able to retrieve clean biogeographic patterns by omitting all groups of easily dispersing littoral to marine turtles from consideration and thereby concentrating our efforts on discerning patterns among slowly dispersing, terrestrial to freshwater aquatic turtles. Only through the omission of saltwater tolerant turtles can clear biogeographic provinces be established throughout much of the Mesozoic, because numerous clades previously thought to have a global distribution (e.g., Pleurodira, Cryptodira) are shown to have wide distribution because of their marine representatives only. Although the phylogenetic relationships of littoral to marine turtles remain poorly resolved, the shear number of clades that likely invaded saltwater habitat independently from one another further highlights the ecological flexibility of turtles throughout their evolutionary history.

### New insights into the phylogeny of fossil turtles

The great amount of new fossil turtle material described above not only has a significant impact by better documenting the temporal and spatial distribution of numerous groups of turtles, but also by further resolving their phylogenetic relationships. Among many new conclusions, the recognition of an extended turtle stem lineage is among the most significant advancements (e.g., [8, 10–12, 25, 26, 41–43]. Early cladistic hypotheses had presumed that the cryptodiran and pleurodiran stem lineage split from one another during the Triassic [83] and various basal turtles groups now thought to be stem turtles were placed along the cryptodiran stem lineages ([5, 7] and references therein). This implies that both groups diverged from one another in sympatry and that cryptodires and pleurodires came to “dominate” the northern and southern hemispheres, respectively, during the later Mesozoic through non-random extinction [6]. Our model, by contrast, more parsimoniously asserts the primary, global presence of turtles throughout the Late Triassic and Early Jurassic and the secondary breakup of crown turtles during the Middle Jurassic due to the breakup of Pangaea.

The phylogeny of fossil turtles is still not fully resolved, but we expect our biogeographic model to prevail because we focused on efforts on tracing the biogeographic histories of seven uncontroversial clades of turtles. A broad sample of fossil turtles nevertheless remains with uncertain phylogenetic affiliations, because they are based on fragmentary remains and therefore lack diagnostic characteristics. If these problematic taxa are freshwater aquatic or terrestrial, our model predicts that future work will shown them to be 1) basal turtles that diversified prior to the breakup of Pangaea, or 2) herein unaccounted for basal turtles that survived the breakup of Pangaea, or 3) representatives of the seven primary clades of turtles outlined herein (or various clade combinations thereof) on the appropriate land mass. The vast majority of these unresolved taxa are from the Late Jurassic and Early Cretaceous, but the enigmatic *Kallokibotion bajazidi* Nopcsa 1923 [133] is an exception. In the vast majority of recent phylogenetic hypotheses (e.g., [8–12, 25, 26, 41–43, 47]), this Late Cretaceous taxon is placed in a similar phylogenetic “level” as Helochelydridae, Sichuanchelyidae, and Meiolaniformes, but is never directly associated with any of these clades. The phylogenetic position of this taxon is somewhat ambiguous because it is known from relatively poorly preserved material [133, 134]. However, a literal interpretation of current analyses would suggest that *K. bajazidi* is yet another isolated lineage of basal turtles that survives along the eastern edge of Europe.

## Conclusions

We here describe a new basal turtle, *Sichuanchelys palatodentata*, on the basis of seven mostly subadult, partial skeletons collected from the early Late Jurassic (Oxfordian) Shishugou Formation of Wucaiwan, Xinjiang, China. The new turtle greatly resembles the previously named species *Sichuanchelys chowi* from the Middle Jurassic of Sichuan, China by having a broad nuchal emargination that is delimited by peripheral II, vertebral scutes that are broader than long, marginals that are restricted to the peripherals, a ligamentous bridge, a broad plastron, a pair of mesoplastra with a midline contact, a short midline contact of the epiplastra, and anteroposteriorly short extragular scutes, but differs by consistently exhibiting a contact between vertebral I and marginal II. Unlike *Sichuanchelys chowi*, the available material of *Sichuanchelys palatodentata* includes beautifully preserved skulls that notably exhibit frontals that are excluded from the orbits, an elongated jugal that nearly contacts the quadrate posteriorly, posteriorly extended squamosals that partially roof the neck region, pterygoid teeth, a closed interpterygoid vacuity, formed foramina posterius canalis carotici palatinum, a visible, but fused basicranial joint, a prootic that is visible in ventral view, and an anteriorly placed canalis stapedio-temporalis. Phylogenetic analysis solidly places *Sichuanchelys chowi* and *Sichuanchelys palatodentata* in a clade with the Late Cretaceous (Maastrichtian) *Mongolochelys efremovi* to form the Asian clade Sichuanchelyidae. This clade is placed outside crown group Testudines and forms a paraphyletic grade with the clades Meiolaniformes and Helochelydridae, which are inferred to be dominantly terrestrial and present as well by the Middle Jurassic.

A global review of the fossil record of turtles reveals that the early history of the three primary lineages of crown turtles, Paracryptodira, Pan-Pleurodira, and Pan-Cryptodira, can be traced back to the Middle Jurassic of Euramerica, Gondwana, and Asia, respectively. External evidence reveals that the North Atlantic and Turgai Strait originated at that time. The origin of the two primary lineages of Pleurodira, Pan-Chelidae and Pan-Pelomedusoides, similarly coincides with the emergence of a large desert zone that subdivided Gondwana. The primary divergence of crown turtles was therefore driven by vicariance to the available terrestrial habitat.

Three persistent lineages of basal turtles, Helochelydridae, Meiolaniformes, and Sichuanchelyidae, show geographic and temporal distributions that overlap with those of Paracryptodira, Pan-Chelidae, and Pan-Cryptodira, respectively, but given the lack of consensus on their phylogenetic relationships, it is unclear if this is also a result of vicariance, or differential extinction leading to a vicariance-like distributional pattern.

The vicariant pattern that is apparent among early representatives of crown turtles is secondarily obliterated by extensive dispersal of continental turtles, especially of cryptodires from Asia to all other habitable continents, the invasion of marine habitats by multiple lineages of pleurodires and cryptodires, and through extinction, particularly that of paracryptodires.

## Methods

We analyzed the phylogenetic relationship of *Sichuanchelys chowi* (as described by [16]) and *Sichuanchelys palatodentata* n. sp. by integrating them into the phylogenetic analysis of Zhou and Rabi [12] that builds on the previous matrices of e.g., [8, 9, 42, 47]. The character/taxon matrix was further modified through the addition of the helochelydrids *Naomichelys speciosa* (as described by [101]), *Helochelydra nopcsai* Lapparent de Broin and Murelaga, 1999 (as described by [100]), and the basal turtle *Spoochelys ormondea* Smith and Kear, 2013 [135], through the modification of one character and the addition of six new characters that help establishing the monophyly of helochelydrid and sichuanchelyid turtles, and through the modification of the scoring for various taxa based on personal observations of relevant material. The final matrix consists of 244 characters for a total of 113 terminal taxa. The full list of characters and changes and the character/taxon matrix are provided in Additional files 1 and 2, respectively.

A heuristic search was performed on the dataset in TNT [136] using the tree-bisection reconnection swapping algorithm with 1000 random addition sequence replicates and ten trees saved per replicate. A total of 30 characters that form morphoclines were run ordered (i.e., 7, 19, 27, 39, 41, 48, 50, 57, 75, 76, 82, 88, 109, 110, 114, 119, 122, 125, 126, 127, 146, 147, 149, 164, 176, 198, 199, 217, 218, 231 [as numbered in Mesquite, starting from 1]). A molecular backbone constraint was implemented in the search by forcing the relationships of crown-turtles to the emerging molecular consensus ([14, 84]; see Additional file 1 for topology). All fossil taxa were allowed to freely float within the constrained topology. The pruned strict consensus is provided in Fig. 8. Standard bootstrap values were calculated with 1000 replicates. The strict consensus tree including all wildcard taxa, a list of synapomorphies, and the results of standard bootstrapping are provided in Additional file 3.

It is important to evaluate the biogeographic history of a group with reference to a phylogenetic hypothesis, but the uncritical reliance on one particular topology can lead to spurious results. We note two difficulties in regards to turtles. First, the vast majority of turtle fossils are fragmentary, but most can nevertheless be assigned with confidence to various clades using the surface texture of their shell [91]. However, even though these fragments are essential in documenting the rich biogeographic history of turtles [45], at least to the level for which they are diagnostic, it is impractical to integrate them into global phylogenetic analyses. All currently available phylogenetic hypotheses therefore lack a substantial amount of pivotal data. The second issue we note for turtles is that the monophyly of the majority of groups is not controversial, but that the interrelationship of these clades is still up for debate. As such, biogeographic scenarios drafted from two different phylogenetic hypotheses will differ substantial at the base, but only little towards the tips. Though reasonable, the phylogenetic hypothesis we retrieve herein is affected by the same problems.

We therefore herein attempt to build a descriptive biogeographic history of turtles that utilizes all available fossil and phylogenetic data and that can be tested in the future through more explicit means. For this purpose we establish a topology that summarizes the current consensus (Fig. 9) among morphological phylogenies and that is explicitly justified throughout the main body of the text. We then establish biogeographic patterns among turtles by explicit reference to the following literature: Pan-Chelidae [53, 68, 137]; Pan-Pelomedusoides ([59, 60, 68], with additions from [61, 62, 69, 138], and figured material present in [66]); Paracryptodira ([46, 76, 79], with additions from [77, 81, 139, 140]); Pan-Cryptodira/Cryptodira ([16, 46, 84, 85, 87, 141], with additions from [86, 107]); Meiolaniformes ([5, 68]; with additions from [26, 41, 43, 98, 99]); Helochelydridae [100]; and Sichuanchelyidae [16, 102].

Our analysis rigorously distinguishes between freshwater aquatic to terrestrial turtles on the one side, and littoral to marine turtles on the other side, as inferred from the depositional setting from which taxa were collected. Figure 10 summarizes the biogeographic distribution of the primary clades of freshwater aquatic and terrestrial turtles highlighted in Fig. 9. All paleogeographic maps were created by electronically modifying maps freely available online [142].

## Additional files

Additional file 1: Character list and changes. (PDF 191 kb)
Additional file 2: Character taxon matrix. (NEX 144 kb)
Additional file 3: Results of the phylogenetic analysis. (PDF 392 kb)

## Abbreviation

IVPP: Institute of Vertebrate Paleontology and Paleoanthropology, Beijing
