# Supplementary material for: A toothed turtle from the Late Jurassic of China and the global biogeographic history of turtles
Source: BMC Evol Biol. 2016 Oct 28;16:236. doi: 10.1186/s12862-016-0762-5 (PMC5084352; doi:10.1186/s12862-016-0762-5)
Supplement: Additional file 1: — Character list and changes. (PDF 191 kb) [file 12862_2016_762_MOESM1_ESM.pdf]

A Toothed Turtle from the Late Jurassic of China and the Global Biogeographic History of Turtles

Walter G. Joyce[1*], Márton Rabi[2], James M. Clark[3], Xing Xu[4]

Additional file 1: Character list and changes

A. Character List

B. List of taxa excluded from analysis

C. List changes to character/taxon matrix

D. Molecular backbone constraint

A. Character List

Characters 1–124: see Zhou and Rabi (2015)

Character 125: Number of suprapygals: 0 = none; 1 = one; 2 = two; 3 = three or more (modified from Zhou and Rabi (2015) by rearranging character states to allow ordering).

Character 126: Number of cervicals: 0 = none; 1 = one; 2 = two or more (modified from Zhou and Rabi (2015) by rearranging character states to allow ordering).

Characters 127–230: see Zhou and Rabi (2015)

Characters 231: Nuchal emargination: 0 = absent or indistinct; 1 = present, excludes peripheral I; 2 = present, includes peripheral I; 3 = present, includes peripheral II (modified from Zhou and Rabi (2015) by adding character state 3)

Characters 232–238: see Zhou and Rabi (2015)

Characters 239: antrum postoticum: 0 = antrum postoticum enlarged and laterally enclosed; 1 = antrum postoticum enlarged, but not enclosed laterally (new character)

Characters 240: jugal/quadrate contact: 0 = jugal clearly not in contact with quadrate, quadratojugal broad; 1 = jugal nearly or clearly in contact with quadrate, quadratojugal reduced (new character)

Characters 241: parabasisphenoid decorated by anteroposterior ridges: 0 = absent; 1 = present

Characters 242: entoplastral scute: 0 = absent; 1 = present (new character)

Characters 243: secondary pair of basioccipital tubercles formed by pterygoid: 0 = absent; 1 = present (new character)

Characters 244: shell covered by highly distinct tubercles: 0 = absent; 1 = present (new character)

B. List of taxa excluded from analysis (relative to Zhou et al., 2014)

Santanachelys gaffneyi

Comment: This taxon is poorly described and we were unable to make first hand observations.

Simosaurus gaillardoti

Comment: This sauropterygian was placed within Testudinata in our first analysis. Due to this obvious artefact and the fact that most of the characters employed here are inapplicable for this taxon, we omitted this taxon.

Chengyuchelys

Comment: The content of this poorly known taxon is unclear (Tong et al. 2012). It furthermore acts in most analyses as a wild card taxon. We therefore omitted a priori.

Dinochelys whitei

Comment: We suspect that this taxon is a juvenile of *Glyptops plicatulus*.

Ordosemys skull

Comment: This terminal generally acts as wildcard taxon, significantly increases computational time, and is not relevant to the present discussion. Mesozoic stem-cryptodires from Asia are otherwise represented by many other OUTs, including *Ordosemys leios*.

C. List changes to character/taxon matrix

We undertook a number of changes to the scoring of Mongolochelys efremovi and Kallokibotion bajadizi based on personal observations (WGJ + MR) of relevant material. The following changes were implemented:

***Mongolochelys efremovi*:**

Nasal B:	0 --> 0&1
Prefrontal D:	0 --> 1
Parietal C:	? --> 0
Parietal G:	? --> 1
Vomer E:	1 --> 0
Pterygoid I :	0/1 --> 1
Pterygoid L:	0 --> ?
Supraoccipital A:	1 --> 0

Supraoccipital B: 1 --> 0

Basisphenoid B: 0 --> 1

Suprapygal A: 0 --> 2

Cervical A: 0 --> 1

Vertebral C: 0 --> –

Epiplastron B: ? --> 1

Extragular B: 0 --> 1

Hypoischium: ? --> 1

***Kallokibotion bajazidi*:**

Parietal C 0 --> ?

Vomer E: 1 --> ?

Quadrate F: 3 --> ?

Supraoccipital A: 1 --> ?

Basisphenoid B: 0 --> ?

Suprapygal A: 1 --> 2

Hypoplastron B: 2 --> ?

Extragular B: 0 --> 1

Cleithrum A: 1 --> 2

D. Molecular backbone constraint

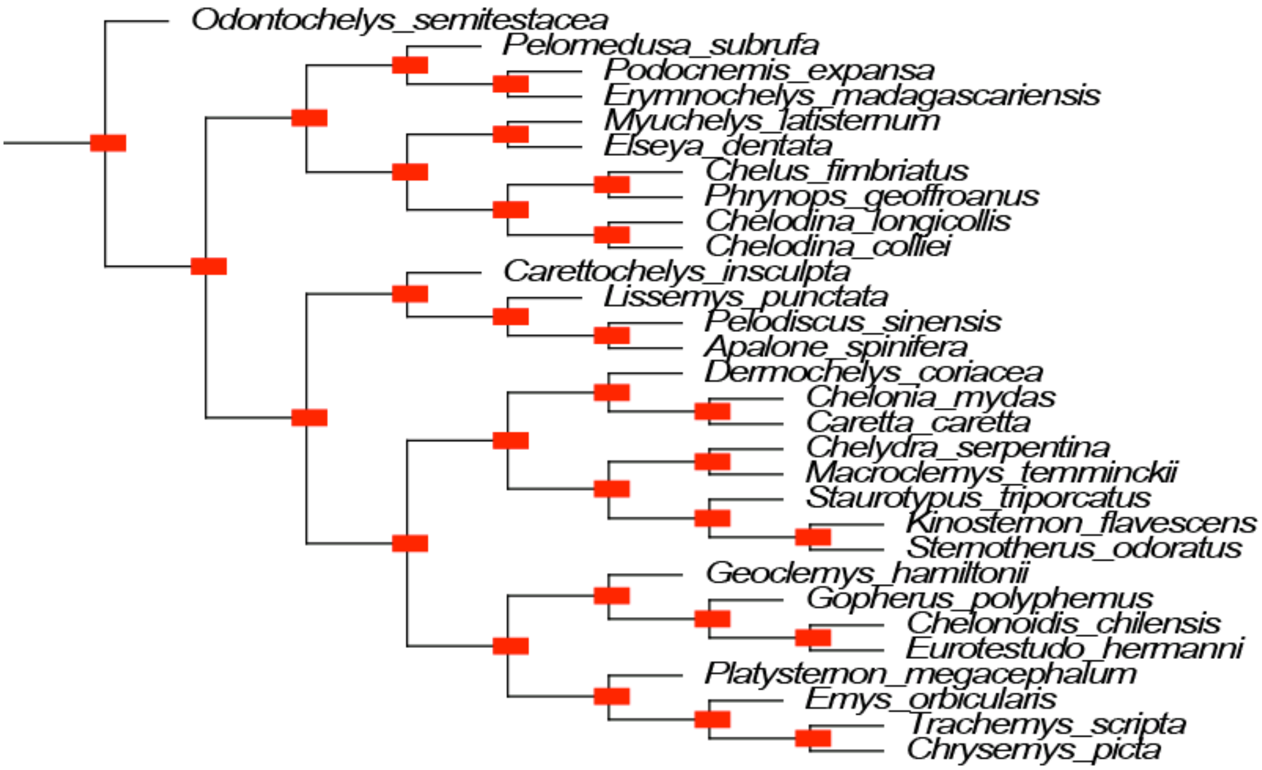

References

Tong H, Danilov I, Ye Y, Ouyang H, Peng G. Middle Jurassic turtles from the Sichuan Basin, China: a review. Geol Mag. 2012;149:675–95.

Zhou C-F, & Rabi M. A sinemydid turtle from the Jehol Biota provides insights into the basal divergence of crown turtles. Sci. Rep. 2015;5:16299.

Zhou C-F, Rabi M, Joyce WG. A new specimen of *Manchurochelys manchoukuoensis* from the Early Cretaceous Jehol Biota of Chifeng, Inner Mongolia, China and the phylogeny of Cretaceous basal eucryptodiran turtles. BMC Evol Biol. 2014;14:77.
