# Supplementary material for: A toothed turtle from the Late Jurassic of China and the global biogeographic history of turtles
Source: BMC Evol Biol. 2016 Oct 28;16:236. doi: 10.1186/s12862-016-0762-5 (PMC5084352; doi:10.1186/s12862-016-0762-5)

A Toothed Turtle from the Late Jurassic of China and the Global Biogeographic History of Turtles

Walter G. Joyce[1*], Márton Rabi[2], James M. Clark[3], Xing Xu[4]

Additional file 3: Results of the phylogenetic analysis

A. Strict consensus topology resulting from 550 most parsimonious trees with 960 steps including all wildcard taxa

B. Synapomorphies common to 550 trees

C: Alternative positions of wildcard taxa

D. Frequency differences values of standard bootstrap resampling of 1000 replicates

E. Frequency differences values of standard bootstrap resampling of 1000 replicates with 5 wild card taxa excluded

A. Strict consensus topology resulting from 550 most parsimonious trees with 960 steps including all wildcard taxa

Numbers denote nodes.

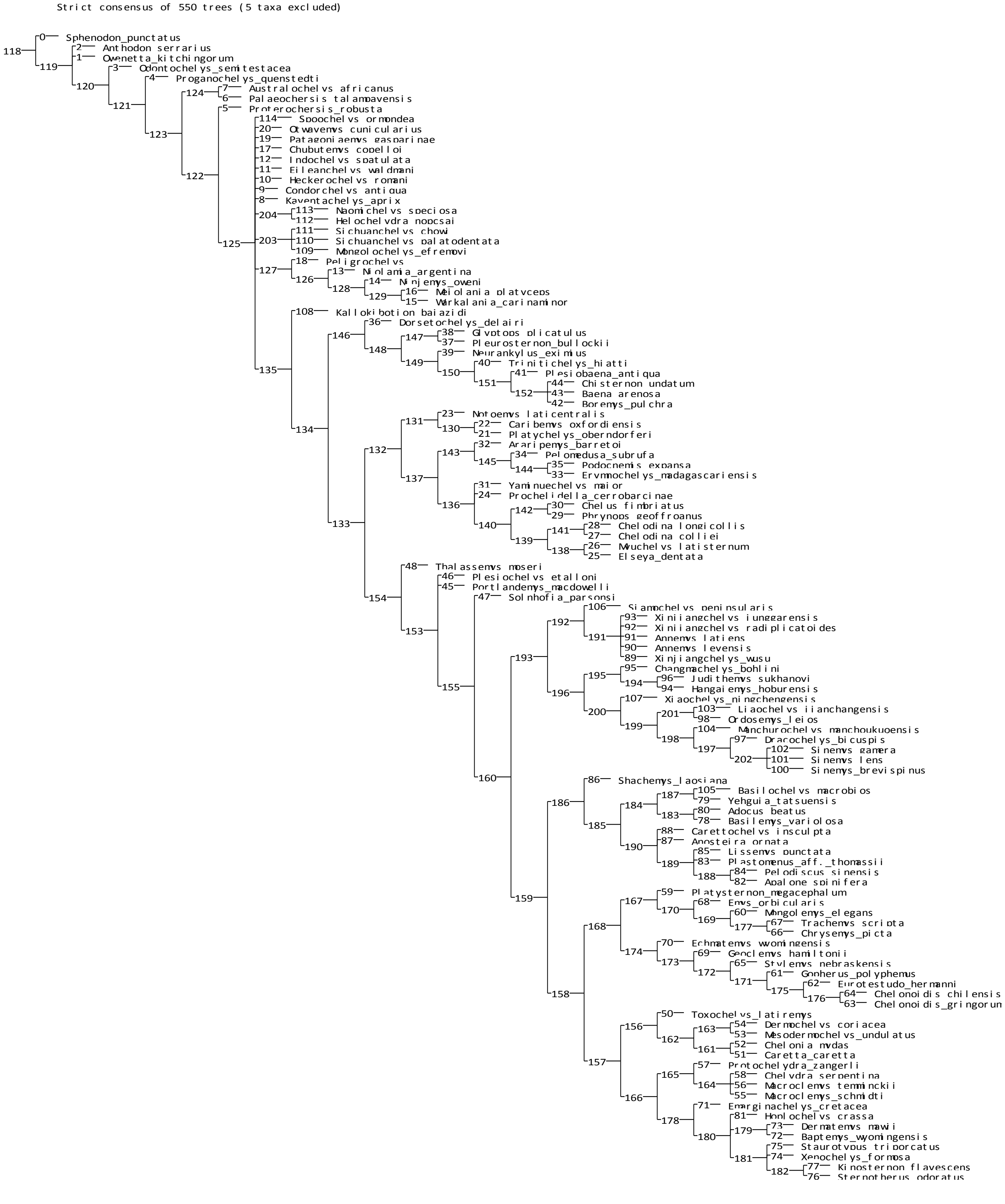

B. Synapomorphies common to 550 trees

Numbers refer to nodes in consensus tree above.

Sphenodon_punctatus :
All trees:
No autapomorphies:

Owenetta_kitchingorum :
All trees:
Prefrontal_A (3): medial_contact_on_dorsal_skull_roof_absent --> medial_contact_on_dorsal_skull_roof_present

Anthodon_serrarius :
All trees:
Prefrontal_E (7): absent --> prefrontal_heavily_sculptured_present
Pterygoid_B (56): basipt_process_present_and_movable_articulation --> basipt_process_present_and_sutured_articulation
Some trees:
Humerus_E* (203): length_of_the_humerus_more_than_two_times_the_width_of_the_proximal_end --> length_of_the_humerus_two_times_or_less_than_the_width_of_the_proximal_end

Odontochelys_semitestacea :
All trees:
Cervical_vertebra_A (169): anterior_end_of_the_centrum --> position_of_transverse_processes_middle_of_the_centrum
Manus_A (215): most_digits_with_two_shortened_phalanges --> most_digits_with_three_elongate_phalanges

Proganochelys_quenstedti :
All trees:
Prefrontal_E (7): absent --> prefrontal_heavily_sculptured_present
Prootic_A* (71): dorsal_exposure_large --> dorsal_exposure_reduced_or_absent
Caudal_A (188): absent --> tail_club_present
Pes_B (219): metatarsal_V_functions_as_a_tarsal --> metatarsal_V_functions_as_true_metatarsal
Some trees:
Humerus_E* (203): length_of_the_humerus_more_than_two_times_the_width_of_the_proximal_end --> length_of_the_humerus_two_times_or_less_than_the_width_of_the_proximal_end

Proterochersis_robusta :
All trees:
Xiphiplastron_A (149): distinct_anal_notch_absent --> present
Pelvis_A (204): pelvis-shell_attachment_by_ligaments --> ischium_attached_to_plastron_by_a_broad_suture

Palaeochersis_talampayensis :
All trees:
Maxilla_D*_ (38): labial_and_lingual_ridge_present --> Triturating_surface_with_only_labial_ridge_present

Australochelys_africanus :
All trees:
No autapomorphies:

Kayentachelys_aprix :
Some trees:
Nasal_C (2): dorsal_exposure_of_nasal_large --> greately_reduced_relative_to_that_of_all_other_elements
Pterygoid_A (55): absent --> pterygoid_teeth_present
Dentary_A (107): medial_contact_of_dentaries_fused --> sutured_only
Suprapygal_A* (124): two_elements --> one_element
Plastron_B (132): present --> central_plastral_fontanella_absent
Nuchal_emargination (230): absent_or_indistinct, or present,_excludes_peripheral_1 --> deep_and_involves_peripheral_1

Condorchelys_antiqua :
All trees:
No autapomorphies:

Heckerochelys_romani :
All trees:
Epiplastron_A (141): epiplastra_and_entoplastron_narrow_and_elongate_absent --> present

Some trees:
Pterygoid_I (63): vertical_flange_on_lateral_process_absent --> vertical_falnge_on_lateral_process_present
Musk_ducts_A* (119): absent --> present
Entoplastron_B (137): size_of_posterior_entoplastral_process_long --> short
Entoplastron_C (138): absent --> distinct_posterolateral_entoplastral_process_present
Cervical_articulation_A (172): formed --> not_formed

Eileanchelys_waldmani :
Some trees:
Antrum_postoticum_A (49): incipient --> fully_developed
Peripheral_gutter (228): peripheral_gutter_absent_or_only_anteriorly_developed --> peripheral_gutter_extensively_developed_along_anterior_and_bridge_peripherals
Nuchal_emargination (230): absent_or_indistinct, or present,_excludes_peripheral_1 --> deep_and_involves_peripheral_1

Indochelys_spatulata :
Some trees:
Entoplastron_A (136): absent --> anterior_entoplastral_process_present
Neural_number (223): less_than_9_elements --> 9_elements

Niolamia_argentina :
All trees:
Cranial_scute_C* (91): yes --> Scute_X_much_smaller_than_D_scute_no
Cranial_scute_N* (102): H_scute_present --> absent
Some trees:
Cranial_scute_K* (99): A_scute_comparable_in_size_to_B_scute --> A_scute_small_A_scute_very_large

Ninjemys_oweni :
All trees:
Cranial_scute_F* (94): low --> D_scute_high

Warkalania_carinaminor :
All trees:
No autapomorphies:

Meiolania_platyceps :
All trees:
Cranial_scute_E* (93): Scutes_A,_B,_and_C_forming_a_continuous_posterolateral_shelf_yes --> no

Chubutemys_copelloi :
Some trees:
Vomer_B (41): vomer-pterygoid_contact_in_palatal_view_present --> absent,_medial_contact_of_palatines_present
Supraoccipital_A (66): crista_occipitalis_poorly_developed --> protruding_significantly_posterior_to_the_foramen_magnum

Peligrochelys :
Some trees:
Canalis_caroticum_F*_ (87): Arteria_palatina_enters_the_skull_through_the_interpterygoid_vacuity_or_intrapterygoid_slit --> through_foramen_posterius_canalis_carotici_palatinum_or_split_of_branches_enclosed_in_skull

Patagoniaemys_gasparinae :
Some trees:
Cervical_vertebra_A (169): position_of_transverse_processes_middle_of_the_centrum --> anterior_end_of_the_centrum
Dorsal_rib_A (184): length_first_thoracic_rib_long,_extends_full_legth_of_first_costal_and_may_contact_peripherals --> intermediate,_in_contact_with_axillary_buttresses

Otwayemys_cunicularius :
Some trees:
Mesoplastron_A (146): 1_or_2_pairs_of_meso_with_medial_contact, or 1_reduced_pair --> absent
Hypoplastron_B* (148): Inguinal_buttress_terminates_on_peripheral_8 --> 6
Extragular_C (156): absent --> anterior_plastral_tuberosities_present
Abdominal_A (163): present,_with_medial_contact --> present,_medial_contact_absent
Dorsal_rib_A (184): length_first_thoracic_rib_long,_extends_full_legth_of_first_costal_and_may_contact_peripherals --> intermediate,_in_contact_with_axillary_buttresses

Platychelys_oberndorferi :

All trees:
Musk_ducts_A* (119): absent --> present
Supramarginal_A (126): absent --> partial_row_present
Ilium_B (209): positioned_on_costals_only --> iliac_scar_extends_from_costals_onto_the_peripherals_and_pygal

Caribemys_oxfordiensis :
All trees:
Pubis_B* (207): cartilaginous_or_absent --> Epipubis_process_osseous_or_calcified

Notoemys_laticentralis :
All trees:
No autapomorphies:

Prochelidella_cerrobarcinae :
All trees:
No autapomorphies:

Elseya_dentata :
All trees:
Maxilla_D*_ (38): Triturating_surface_with_only_labial_ridge_present --> labial,_lingual_and_accesory_ridges_present

Myuchelys_latisternum :
All trees:
Basisphenoid_B (77): paired_pits_on_ventral_surface_absent --> present

Chelodina_colliei :
All trees:
No autapomorphies:

Chelodina_longicollis :
All trees:
No autapomorphies:

Phrynops_geoffroanus :
All trees:
Peripheral_gutter (228): peripheral_gutter_absent_or_only_anteriorly_developed --> peripheral_gutter_extensively_developed_along_anterior_and_bridge_peripherals

Chelus_fimbriatus :
All trees:
Nasal_A (0): present --> absent
Prefrontal_C (5): prefrontal-palatine_contact_absent --> prefrontal-palatine_contact_present
Premaxilla_B (32): fusion_of_premaxilla_absent --> present
Vomer_D (43): absent --> vomer-premaxilla_contact_present
Carapace_B (109): tricarinate_carapace_absent --> present_and_pronounced
Costal_B (121): medial_contact_of_up_to_three_posterior_costals_present --> medial_contact_of_posterior_costals_absent
Costal_C (122): absent,_costals_fully_or_almost_fully_ossified,_fontanelles_abs_or_red --> present
Xiphiplastron_B (150): xiphiplastra_narrow_absent --> present
Extragular_B (155): medial_contact_of_extragulars_absent --> present,_contacting_one_another_posterior_to_gulars
Extragular_C (156): absent --> anterior_plastral_tuberosities_present
Extragular_D* (157): Only_in_the_epiplastra --> Reach_the_entoplastron
Humeral_B* (160): Humero-pectoral_sulcus_only_in_the_hyoplastra --> humero-pectoral_sulcus_crossing_the_entoplastron
Cervical_vertebra_B (170): ventral_keels_absent_or_slightly_developed_in_all_vertebrae --> ventral_keels_more_developed_on_posterior_vertebrae
Cervical_vertebra_C (171): cervical_centrum_8<7_absent --> present

Yaminuechelys_maior :
All trees:
Carapace_D_* (111): Sculpturing_of_the_shell_absent --> present
Nuchal_emargination (230): absent_or_indistinct, or present,_excludes_peripheral_1 --> deep_and_involves_peripheral_1
Some trees:
Squamosal_B (25): squamosal-supraoccipital_contact_absent --> present
Nuchal_C* (115): longer_than_wide_or_as_long_as_wide --> wider_than_long
Ilium_B (209): positioned_on_costals_only --> iliac_scar_extends_from_costals_onto_the_peripherals_and_pygal

Araripemys_barretoi :
All trees:
Jugal_B (20): jugal_participation_to_upper_temporal_rim_absent --> present
Pterygoid_G (61): medial_contact_of_pterygoids_present --> absent
Carapace_B (109): tricarinate_carapace_absent --> present_and_pronounced
Carapace_D_* (111): Sculpturing_of_the_shell_absent --> present
Costal_C (122): absent,_costals_fully_or_almost_fully_ossified,_fontanelles_abs_or_red --> present
Entoplastron_D (139): entoplastron_V-shaped_absent --> present
Epiplastron_A (141): epiplastra_and_entoplastron_narrow_and_elongate_absent --> present
Mesoplastron_A (146): 1_reduced_pair --> absent
Hypoplastron_A (147): peripheral_and_costal_V --> inguinal_buttresses_contact_peripherals_only
Hypoplastron_B* (148): Inguinal_buttress_terminates_on_peripheral_8 --> 7
Extragular_A (154): present --> absent
Abdominal_A (163): present,_with_medial_contact --> present,_medial_contact_absent
Posterior_plastral_fontanelle (222): posterior_plastral_fontanella_between_the_xiphiplastra_and/or_the_hypoplastra:__absent_in_adult_stage --> retained_in_adults
Neural_number (223): less_than_9_elements --> 9_elements
First_vertebral (227): vertebral_1_does_not_enter_anterior_margin_of_carapace --> enters_anterior_margin
Nuchal_emargination (230): absent_or_indistinct, or present,_excludes_peripheral_1 --> broad,_involved_peripheral_II
Cruciform_plastron (232): absent --> present
Some trees:
Plastron_A (131): connection_between_carapace_and_plastron_osseous --> ligamentous

Erymnochelys_madagascariensis :
All trees:
Hyoplastron_B* (145): terminates_on_peripheral_3 --> Axillary_buttress_terminates_on_peripheral_2_or_1
Extragular_D* (157): Only_in_the_epiplastra --> Reach_the_entoplastron

Pelomedusa_subrufa :
All trees:
No autapomorphies:

Podocnemis_expansa :
All trees:
Maxilla_A* (36): do_not_contact_each_other_in_ventral_view --> contacts_each_other_in_ventral_view
Maxilla_E*_ (39): Accesory_ridge_on_maxilla_present_all_along_the_triturating_surface --> accessory_ridge_only_in_some_sectors_of_the_triturating_surface
Pterygoid_H (62): pterygoid_contribution_to_foramen_palatinum_posterius_present --> absent

Dorsetochelys_delairi :
All trees:
Basisphenoid_B (77): paired_pits_on_ventral_surface_absent --> present

Pleurosternon_bullockii :
All trees:
Suprapygal_A* (124): two_elements --> one_element
Cervical_A (125): one_cervical_present --> cervicals_absent,_carapacial_scutes_otherwise_present
Gular_A (153): one_pair --> only_one_scute

Glyptops_plicatulus :
All trees:
Premaxilla_E (35): distinct,_medial_premaxillary_hook_along_the_labial_margin_absent --> present
Maxilla_D*_ (38): Triturating_surface_with_only_labial_ridge_present --> labial_and_lingual_ridge_present
Some trees:
Nuchal_emargination (230): absent_or_indistinct --> present,_excludes_peripheral_1

Neurankylus_eximius :
All trees:
No autapomorphies:

Trinitichelys_hiatti :
All trees:
No autapomorphies:

Plesiobaena_antiqua :

Some trees:
Cervical_articulation_A (172): not_formed --> formed

Boremys_pulchra :
All trees:
No autapomorphies:

Baena_arenosa :
All trees:
Parietal_H* (18): moderate,_f.s.t._but_not_entire_processes_trochlearis_exposed_in_dorsal_view --> absent_or_weak,_foramen_stapedio-temporale_concealed_in_dorsal_view
Cranial_scutes_A* (89): absent --> present
Nuchal_C* (115): wider_than_long --> longer_than_wide_or_as_long_as_wide
Mesoplastron_A (146): 1_or_2_pairs_of_meso_with_medial_contact --> 1_reduced_pair
Some trees:
Suprapygal_A* (124): two_elements --> one_element
Caudal_D* (191): posterior_caudal_vertebrae_procoelous_or_platycoelous --> posterior_caudal_vertebrae_opisthocoelous

Chisternon_undatum :
Some trees:
Cervical_articulation_A (172): not_formed --> formed

Portlandemys_macdowelli :
All trees:
No autapomorphies:

Plesiochelys_etalloni :
Some trees:
Pterygoid_F (60): foramen_palatinum_posterius_present --> present,_but_open_laterally
Pterygoid_J* (64): reaching_the_exoccipitals --> not_reaching_the_exoccipitals
Cervical_A (125): one_cervical_present --> more_than_one_cervical_present

Solnhofia_parsonsi :
All trees:
Maxilla_C*_ (37): Secondary_palate_formed_by_premaxilla,_maxilla,_and_vomer,_palatines_not_contacting_in_midline_absent --> formed_by_premaxilla,_maxilla,_and_vomer,_palatines_not_contacting_in_midline_present
Vomer_B (41): vomer-pterygoid_contact_in_palatal_view_present --> absent,_medial_contact_of_palatines_present
Pterygoid_I (63): vertical_falnge_on_lateral_process_present --> vertical_flange_on_lateral_process_absent
Suprapygal_A* (124): two_elements --> more_than_2_elements
Epiplastron_A (141): epiplastra_and_entoplastron_narrow_and_elongate_absent --> present
Nuchal_posterior_edge (234): less_than_3_times_longer_than_the_lateral_edge --> more_than_3_times_longer

Thalassemys_moseri :
All trees:
Vomer_B (41): vomer-pterygoid_contact_in_palatal_view_present --> absent,_medial_contact_of_palatines_present
Prootic_A* (71): dorsal_exposure_large --> dorsal_exposure_reduced_or_absent
Some trees:
Pterygoid_F (60): foramen_palatinum_posterius_present --> present,_but_open_laterally
Opisthotic_C (74): present,_with_an_incipient_enclosed_middle_ear_region --> present,_but_modified_with_a_enclosed_middle_ear_region
Dorsal_rib_A (184): length_first_thoracic_rib_long,_extends_full_legth_of_first_costal_and_may_contact_peripherals --> intermediate,_in_contact_with_axillary_buttresses

Toxochelys_latiremys :
All trees:
Anal_A (164): only_cover_parts_of_the_xiphiplastra --> anteromedially_overlap_onto_hypoplastra
Cervical_articulation_L* (180): present --> double_articulation_between_7th_and_8th_absent
Chevron_A (192): absent_or_poorly_developed_along_posterior_caudals --> present_on_nearly_all_caudals

Caretta_caretta :
All trees:
Quadrate_H*_ (52): Processus_trochlearis_oticum_formed_by_a_great_contribution_of_quadrate --> small_contribution_of_the_quadrate
Peripheral_A (118): 11_pairs --> more_than_11_pairs

Chelonia_mydas :
All trees:
Premaxilla_E (35): distinct,_medial_premaxillary_hook_along_the_labial_margin_absent --> present
Maxilla_D*_ (38): Triturating_surface_with_only_labial_ridge_present, or labial_and_lingual_ridge_present --> labial,_lingual_and_accesory_ridges_present

Mesodermochelys_undulatus :
All trees:
Cervical_vertebra_C (171): present --> cervical_centrum_8<7_absent
Cervical_articulation_L* (180): present --> double_articulation_between_7th_and_8th_absent
Articulation_of_posterior_cervical_centra (233): greatly_flattened_outline --> circular_or_subcircular_outline

Dermochelys_coriacea :
All trees:
Carapace_A (108): partially_present --> absent
Peripheral_A (118): 11_pairs --> less_than_10_pairs
Nuchal_emargination (230): present,_excludes_peripheral_1, or deep_and_involves_peripheral_1 --> absent_or_indistinct

Macroclemys_schmidti :
All trees:
Quadrate_F:_incisura_columella_auris (50): present_and_closed,_but_only_enclosing_the_stapes --> partially_closed,_allowing_see_the_columella_auris_in_posterior_view

Macroclemys_temminckii :
All trees:
Supramarginal_A (126): absent --> partial_row_present
Caudal_C* (190): anterior_caudal_vertebrae_procoelous_or_platycoelous --> anterior_caudal_vertebrae_opisthocoelous
Nuchal_emargination (230): absent_or_indistinct --> deep_and_involves_peripheral_1

Protochelydra_zangerli :
All trees:
No autapomorphies:

Chelydra_serpentina :
All trees:
Suprapygal_A* (124): one_element, or two_elements --> more_than_2_elements

Platysternon_megacephalum :
All trees:
Parietal_H* (18): strong,_entire_processus_trochlearis_exposed_in_dorsal_view --> absent_or_weak,_foramen_stapedio-temporale_concealed_in_dorsal_view
Quadratojugal_B (22): quadratojugal-maxilla_contact_absent --> present
Premaxilla_E (35): distinct,_medial_premaxillary_hook_along_the_labial_margin_absent --> present
Quadrate_F:_incisura_columella_auris (50): present,_but_qu_and_the_op_for_an_angle_less_90_degrees_in_lat_view --> present_and_closed,_but_only_enclosing_the_stapes
Basisphenoid_A (76): rostrum_basisphenoidale_flat --> rod-like,_thick,_and_rounded
Neural_B* (117): regular,_often_hexagonal,_longer_than_wide --> irregular_in_shape,_wider_than_long
Musk_ducts_A* (119): absent --> present
Hyoplastron_B* (145): terminates_on_peripheral_3 --> terminates_on_peripheral_4
Hypoplastron_B* (148): 7 --> 6
Cervical_vertebra_B (170): ventral_keels_more_developed_on_posterior_vertebrae --> ventral_keels_absent_or_slightly_developed_in_all_vertebrae
Caudal_D* (191): posterior_caudal_vertebrae_procoelous_or_platycoelous --> posterior_caudal_vertebrae_opisthocoelous
Chevron_A (192): absent_or_poorly_developed_along_posterior_caudals --> present_on_nearly_all_caudals
Humerus_A* (199): only_a_groove --> Ectepicondylar_foramen_in_a_channel
Humerus_D* (202): lateral_process_not_seen_in_dorsal_view --> lateral_process_seen_in_dorsal_view
Nuchal_emargination (230): absent_or_indistinct --> deep_and_involves_peripheral_1
Tail_length (231): tail_clearly_shorter_than_carapace --> tail_as_long_as_carapace
Nuchal_posterior_edge (234): less_than_3_times_longer_than_the_lateral_edge --> more_than_3_times_longer

Mongolemys_elegans :
All trees:
Pterygoid_H (62): absent --> pterygoid_contribution_to_foramen_palatinum_posterius_present
Basioccipital_A (70): tubercle_absent --> with_two_or_one_ventral_basioccipital_tubercle
Hyoplastron_B* (145): terminates_on_peripheral_3 --> Axillary_buttress_terminates_on_peripheral_2_or_1

Chevron_A (192): absent_or_poorly_developed_along_posterior_caudals --> present_on_nearly_all_caudals
Carotid_canal_split (237): 3 -->
not_enclosed_but_carotid_canal_is_covered_ventrally_from_the_posterior_end_of_the_skull

Gopherus_polyphemus :
All trees:
Humerus_A* (199): only_a_groove --> Ectepicondylar_foramen_in_a_channel

Eurotestudo_hermanni :
All trees:
Prootic_A* (71): dorsal_exposure_large --> dorsal_exposure_reduced_or_absent
Hypoplastron_A (147): peripheral_and_costal_V --> inguinal_buttresses_contact_peripherals_only

Chelonoidis_gringorum :
All trees:
Supramarginal_A (126): absent --> complete_row_present

Chelonoidis_chilensis :
All trees:
Vertebral_C (129): on_neural_V --> sulcus_between_V_3_and_4_on_neural_VI

Stylemys_nebraskensis :
All trees:
Hypoplastron_A (147): peripheral_and_costal_V --> peripherals,_costal_V,_and_costal_VI
Pelvis_A (204): pelvis-shell_attachment_by_ligaments --> ischium_attached_to_plastron_by_a_broad_suture
Pubis_B* (207): cartilaginous_or_absent --> Epipubis_process_osseous_or_calcified

Chrysemys_picta :
All trees:
Pterygoid_D (58): present --> pterygoid-basioccipital_contact_absent
Pectoral_B* (162): antero-posteriorly_developed --> very_short_antero-posteriorly

Trachemys_scripta :
All trees:
Parietal_A (11): absent --> parietal-squamosal_contact_present
Maxilla_E*_ (39): Accesory_ridge_on_maxilla_present_all_along_the_triturating_surface -->
accessory_ridge_only_in_some_sectors_of_the_triturating_surface
Costal_D* (123): absence_of_alternative_short_and_long_ends_in_the_lateral_part_of_the_costals --> presence

Emys_orbicularis :
All trees:
Pterygoid_D (58): present --> pterygoid-basioccipital_contact_absent
Plastron_C (133): plastral_kinesis_absent --> present
Entoplastron_B (137): short --> size_of_posterior_entoplastral_process_long
Xiphiplastron_A (149): present --> distinct_anal_notch_absent

Geoclemys_hamiltonii :
All trees:
Jugal_B (20): jugal_participation_to_upper_temporal_rim_absent --> present
Prootic_A* (71): dorsal_exposure_large --> dorsal_exposure_reduced_or_absent
Carapace_B (109): tricarinate_carapace_absent --> present,_but_only_slightly
Musk_ducts_A* (119): absent --> present

Echmatemys_wyomingensis :
All trees:
Hyoplastron_B* (145): terminates_on_peripheral_3 --> Axillary_buttress_terminates_on_peripheral_2_or_1

Emarginachelys_cretacea :
All trees:
No autapomorphies:

Baptemys_wyomingensis :
All trees:
Hypoplastron_A (147): inguinal_buttresses_contact_peripherals_only --> peripheral_and_costal_V

Dermatemys_mawii :
All trees:

Carapace_B (109): present,_but_only_slightly --> tricarinate_carapace_absent
Xiphiplastron_A (149): distinct_anal_notch_absent --> present
Intergular_A (158): absent --> present
Humeral_B* (160): humero-pectoral_sulcus_crossing_the_entoplastron --> Humero-pectoral_sulcus_only_in_the_hyoplastra
Cervical_vertebra_E* (174): present --> Biconvex_cervical_vertebra_in_the_middle_of_the_neck_absent
Some trees:
Costal_B (121): medial_contact_of_posterior_costals_absent --> medial_contact_of_up_to_three_posterior_costals_present

Xenochelys_formosa :
All trees:
Pterygoid_F (60): foramen_palatinum_posterius_present --> absent
Nuchal_C* (115): wider_than_long --> longer_than_wide_or_as_long_as_wide
Xiphiplastron_A (149): distinct_anal_notch_absent --> present
Nuchal_emargination (230): absent_or_indistinct --> present,_excludes_peripheral_1

Staurotypus_triporcatus :
All trees:
Carapace_B (109): present,_but_only_slightly --> present_and_pronounced
Some trees:
Jugal_B (20): jugal_participation_to_upper_temporal_rim_absent --> present
Cruciform_plastron (232): absent --> present

Sternotherus_odoratus :
Some trees:
Cruciform_plastron (232): absent --> present

Kinosternon_flavescens :
All trees:
Musk_ducts_A* (119): present --> absent
Plastral_kinesis_A* (134): anterior --> anterior_and_posterior
Pubis_B* (207): Epipubis_process_osseous_or_calcified --> cartilaginous_or_absent

Basilemys_variolosa :
All trees:
Extragular_D* (157): Only_in_the_epiplastra --> Reach_the_entoplastron
Inframarginal_B* (166): 3_or_more --> 2
Manus_A (215): most_digits_with_three_elongate_phalanges --> most_digits_with_two_shortened_phalanges
Nuchal_emargination (230): absent_or_indistinct --> deep_and_involves_peripheral_1

Yehguia_tatsuensis :
All trees:
Humeral_B* (160): humero-pectoral_sulcus_crossing_the_entoplastron --> Humero-pectoral_sulcus_only_in_the_hyoplastra

Adocus_beatus :
All trees:
Pectoral_B* (162): antero-posteriorly_developed --> very_short_antero-posteriorly
Illium_E* (212): thelial_process_absent --> present

Hoplochelys_crassa :
All trees:
Carapace_B (109): present,_but_only_slightly --> present_and_pronounced
Epiplastron_B* (142): thick_anterior_border_absent --> thick_anterior_border
Hyoplastron_B* (145): terminates_on_peripheral_3 --> Axillary_buttress_terminates_on_peripheral_2_or_1

Apalone_spinifera :
All trees:
Maxilla_D*_ (38): Triturating_surface_with_only_labial_ridge_present --> labial_and_lingual_ridge_present

Plastomenus_aff._thomassii :
All trees:
No autapomorphies:

Pelodiscus_sinensis :
All trees:

Pterygoid_H (62): absent --> pterygoid_contribution_to_foramen_palatinum_posterius_present

Lissemys_punctata :
All trees:
Pterygoid_I (63): vertical_falnge_on_lateral_process_present --> vertical_flange_on_lateral_process_absent
Hyo-hypoplastron_A* (144): not_fused --> fused
Some trees:
Foramen_jugulare_posterius_B* (85): separated_by_opisthotic_and_or_exoccipital --> separated_from_fenestra_postotica_by_pterygoid

Shachemys_laosiana :
All trees:
Prootic_A* (71): dorsal_exposure_large --> dorsal_exposure_reduced_or_absent
Nuchal_C* (115): wider_than_long --> longer_than_wide_or_as_long_as_wide
Costal_B (121): medial_contact_of_up_to_three_posterior_costals_present --> medial_contact_of_all_costals_present
Cervical_A (125): one_cervical_present --> cervicals_absent,_carapacial_scutes_otherwise_present
Vertebral_B (128): vertebrals_II-IV_narrower_or_as_narrow_as_pleurals --> vertebral_II-IV_broader_than_pleurals

Anosteira_ornata :
All trees:
Nuchal_emargination (230): absent_or_indistinct --> present,_excludes_peripheral_1

Carettochelys_insculpta :
All trees:
Quadratojugal_B (22): quadratojugal-maxilla_contact_absent --> present
Some trees:
Humerus_C* (201): lateral_process_in_the_proximal_end_of_the_humerus --> displaced_from_the_proximal_end,_located_in_the_shaft_of_the_humerus

Xinjiangchelys_wusu :
All trees:
Plastral_scutes_B (152): present --> pronounced_midline_plastral_sulcus_sinuous_absent

Annemys_levensis :
All trees:
Costal_B (121): medial_contact_of_posterior_costals_absent --> medial_contact_of_up_to_three_posterior_costals_present
Vertebral_C (129): on_neural_V --> sulcus_between_V_3_and_4_on_neural_VI
Some trees:
Nuchal_emargination (230): present,_excludes_peripheral_1 --> deep_and_involves_peripheral_1

Annemys_latiens :
Some trees:
Nuchal_emargination (230): present,_excludes_peripheral_1 --> deep_and_involves_peripheral_1

Xinjiangchelys_radiplicatoides :
All trees:
No autapomorphies:

Xinjiangchelys_junggarensis :
All trees:
No autapomorphies:

Hangaiemys_hoburensis :
All trees:
Pterygoid_J* (64): reaching_the_exoccipitals --> not_reaching_the_exoccipitals
Foramen_jugulare_posterius_A* (84): coalescent_with_fenestra_postotica --> separated_from_fenestra_postotica

Changmachelys_bohlini :
All trees:
No autapomorphies:

Judithemys_sukhanovi :
All trees:
Peripheral_gutter (228): peripheral_gutter_extensively_developed_along_anterior_and_bridge_peripherals --> peripheral_gutter_absent_or_only_anteriorly_developed

Dracochelys_bicuspis :
All trees:
Maxilla_D*_ (38): Triturating_surface_with_only_labial_ridge_present --> labial_and_lingual_ridge_present
Basioccipital_A (70): with_two_or_one_ventral_basioccipital_tubercle --> tubercle_absent
Basisphenoid_B (77): present --> paired_pits_on_ventral_surface_absent
Shape_of_costal_3 (225):
costal_3_tapering_towards_the_lateral_side_of_the_shell_or_with_parallel_anterior_and_posterior_borders --> costal_3_broadens_towards_the_lateral_side_of_the_shell
Costal_rib (226): distal_portion_of_costal_ribs_not_visible_within_the_costal --> distal_portion_of_costal_rib_visible_on_the_surface_of_the_costal

Ordosemys_leios :
All trees:
Quadrate_H*_ (52): Processus_trochlearis_oticum_formed_by_a_great_contribution_of_quadrate --> small_contribution_of_the_quadrate
Costal_rib_distal_end (229): costo-peripheral_fontanelles_absent,_distal_end_of_posterior_dorsal_ribs_visible_or_distal_end_of_posterior_costals_narrow_and_surrounded_by_the_peripheral --> distal_end_of_dorsal_rib_not_visible_or_only_within_costo-peripheral_fontanelles_on_the_dorsal_face_of_the_carapace

Sinemys_brevispinus :
Some trees:
Humerus_B* (200): shoulder_present --> shoulder_absent:_pleurodires
Posterior_plastral_fontanelle (222):
posterior_plastral_fontanella_between_the_xiphiplastra_and/or_the_hypoplastra:__absent_in_adult_stage --> retained_in_adults

Sinemys_lens :
All trees:
No autapomorphies:

Sinemys_gamera :
All trees:
No autapomorphies:

Liaochelys_jianchangensis :
All trees:
Costal_B (121): medial_contact_of_posterior_costals_absent --> medial_contact_of_up_to_three_posterior_costals_present
Shape_of_costal_3 (225):
costal_3_tapering_towards_the_lateral_side_of_the_shell_or_with_parallel_anterior_and_posterior_borders --> costal_3_broadens_towards_the_lateral_side_of_the_shell
Costal_rib (226): distal_portion_of_costal_ribs_not_visible_within_the_costal --> distal_portion_of_costal_rib_visible_on_the_surface_of_the_costal

Manchurochelys_manchoukuoensis :
All trees:
Nuchal_emargination (230): present,_excludes_peripheral_1 --> absent_or_indistinct

Basilochelys_macrobios :
All trees:
No autapomorphies:

Siamochelys_peninsularis :
All trees:
Carapace_D_* (111): Sculpturing_of_the_shell_absent --> present
Hyoplastron_B* (145): Axillary_buttress_terminates_on_peripheral_2_or_1 --> terminates_on_peripheral_3
Hypoplastron_B* (148): Inguinal_buttress_terminates_on_peripheral_8 --> 7

Xiaochelys_ningchengensis_ :
All trees:
No autapomorphies:

Kallokibotion_bajazidi :
All trees:
Prefrontal_D (6): reduced --> prefrontal_exposure_large
Premaxilla_A (31): united --> external_nares_divided

Vomer_B (41): vomer-pterygoid_contact_in_palatal_view_present --> absent,_medial_contact_of_palatines_present
Prootic_A* (71): dorsal_exposure_large --> dorsal_exposure_reduced_or_absent
Vertebral_C (129): on_neural_V --> sulcus_between_V_3_and_4_on_neural_VI
Extragular_B (155): medial_contact_of_extragulars_absent --> present,_contacting_one_another_anterior_to_gulars
Humerus_E* (203): length_of_the_humerus_more_than_two_times_the_width_of_the_proximal_end --> length_of_the_humerus_two_times_or_less_than_the_width_of_the_proximal_end

Mongolochelys_efremovi :
All trees:
Squamosal_B (25): squamosal-supraoccipital_contact_absent --> present
Squamosal_C* (26): posterolateral_protuberances_developing_horns_absent --> small_protuberances
Maxilla_D*_ (38): labial_and_lingual_ridge_present --> labial,_lingual_and_accesory_ridges_present
Prootic_A* (71): dorsal_exposure_large --> dorsal_exposure_reduced_or_absent
Cranial_scute_J* (98): A_scute_small_and_not_forming_a_large_shelf_no --> yes
Some trees:
Neural_B* (117): regular,_often_hexagonal,_longer_than_wide --> irregular_in_shape,_wider_than_long
Caudal_B (189): all_centra_amphicoelous --> formed_centra

Sichuanchelys_palatodentata :
Some trees:
Prefrontal_E (7): absent --> prefrontal_heavily_sculptured_present
Pterygoid_A (55): absent --> pterygoid_teeth_present
Fenestra_perilymphatica_A (88): large --> relatively_small
Cranial_scute_F* (94): low --> D_scute_high
Caudal_B (189): all_centra_amphicoelous, or formed_centra --> all_centra_amphicoelous
Humerus_A* (199): Ectepicondylar_foramen_in_a_channel --> only_a_groove
Pterygoid_extension (236): pterygoid_extending_to_posterior_end_of_skull_and_covering_prootic --> _pterygoid_not_extending_to_posterior_end_of_skull_and_covering_prootic

Sichuanchelys_chowi :
All trees:
Plastron_B (132): present --> central_plastral_fontanella_absent
Some trees:
Suprapygal_A* (124): two_elements --> more_than_2_elements

Helochelydra_nopcsai :
All trees:
Quadratojugal_B (22): quadratojugal-maxilla_contact_absent --> present
Supraoccipital_B (67): large_supraoccipital_exposure_to_dorsal_skull_roof_absent --> present

Naomichelys_speciosa :
All trees:
No autapomorphies:

Spoochelys_ormondea :
Some trees:
Pterygoid_C (57): reduced_to_a_paired_foramen_caroticum_laterale --> triangular_in_shape
Cranial_scute_K* (99): A_scute_comparable_in_size_to_B_scute --> A_scute_small_A_scute_very_large
Supramarginal_A (126): absent --> complete_row_present, or partial_row_present
Cervical_vertebra_A (169): position_of_transverse_processes_middle_of_the_centrum, or anterior_end_of_the_centrum --> position_of_transverse_processes_middle_of_the_centrum
Pterygoid_extension (236): pterygoid_extending_to_posterior_end_of_skull_and_covering_prootic --> _pterygoid_not_extending_to_posterior_end_of_skull_and_covering_prootic

Node 119 :
All trees:
No synapomorphies

Node 120 :
All trees:
Basioccipital_A (70): tubercle_absent --> with_two_or_one_ventral_basioccipital_tubercle
Pubis_B* (207): cartilaginous_or_absent --> Epipubis_process_osseous_or_calcified
Hypoischium_A (214): absent --> present
Carotid_canal_split (237): enclosed_but_carotid_canal_is_not_covered_ventrally_from_posterior_edge_of_skull, or 3 --> not_enclosed_in_bone

Node 121 :

All trees:
Teeth_A (105): teeth_present_in_premaxilla,_maxilla,_and_dentary -->
teeth_absent__in_premaxilla,_maxilla,_and_dentary
Carapace_C (110): absent --> present

Node 122 :
All trees:
Ilium_A (208): elongated_iliac_neck_absent --> present

Node 123 :
All trees:
Jugal_A (19): jugal-squamosal_contact_present --> absent
Vomer_C (42): vomerine_and_palatine_teeth_present --> absent
Quadrate_A (46): flooring_of_the_cranioquadrate_space_absent --> by_pt,_but_pt_does_not_cover_the_prootic
Quadrate_B_+_C (47): development_of_the_c.t._shallow,_but_not_developed_antpost -->
shallow,_but_anteroposteriorly_developed
Pterygoid_B (56): basipt_process_present_and_movable_articulation -->
basipt_process_present_and_sutured_articulation
Opisthotic_A (72): loosely_articulated --> tightly_sutured
Opisthotic_C (74): ventral_ridge_on_opisthotic_absent --> present,_with_an_incipient_enclosed_middle_ear_region
Some trees:
Pterygoid_A (55): pterygoid_teeth_present --> absent

Node 124 :
All trees:
Parietal_D (14): overhanging_process_of_the_skull_roof_absent --> present
Opisthotic_B (73): depressions_for_musculature_absent --> present

Node 125 :
All trees:
Supramarginal_A (126): partial_row_present --> absent
Extragular_C (156): anterior_plastral_tuberosities_present --> absent
Some trees:
Musk_ducts_A* (119): absent --> present
Plastron_A (131): connection_between_carapace_and_plastron_osseous --> ligamentous
Cleithrum_A (197): present_and_in_contact_with_the_carapace --> present,_osseous_contact_with_carapace_absent

Node 126 :
All trees:
Squamosal_C* (26): small_protuberances --> big_protuberances_developed_as_horns
Pterygoid_D (58): pterygoid-basioccipital_contact_absent --> present
Cranial_scute_O* (103): Scale_F_formed_by_several_scales --> Scale_F_formed_by_only_one_scale
Some trees:
Pterygoid_C (57): reduced_to_a_paired_foramen_caroticum_laterale --> reduced_to_an_interpterygoid_slit

Node 127 :
All trees:
Pterygoid_B (56): basipt_process_present_and_sutured_articulation --> basipt_process_absent_and_sutured_articulation
Some trees:
Squamosal_C* (26): posterolateral_protuberances_developing_horns_absent --> small_protuberances
Supraoccipital_A (66): crista_occipitalis_poorly_developed -->
protruding_significantly_posterior_to_the_foramen_magnum
Hyomandibular_nerve_A (79): :_path_of_hyomandibular_branch_facial_nerve_through_cranio-
quadrate_space_parallel_to_vena_capitis_lateralis --> independent_to_vena_capitis_lateralis

Node 128 :
All trees:
Maxilla_D*_ (38): labial_and_lingual_ridge_present --> labial,_lingual_and_accesory_ridges_present
Cranial_scute_B* (90): Scute_D_meeting_in_midline_no --> yes
Cranial_scale_P* (104): Scale_J_formed_by_several_scales --> Scale_J_formed_by_only_one_scale

Node 129 :
All trees:
Cranial_scute_J* (98): A_scute_small_and_not_forming_a_large_shelf_no --> yes

Node 130 :
All trees:

Hypoplastron_B* (148): Inguinal_buttress_terminates_on_peripheral_8 --> 7
Ilium_C (210): oval --> shape_of_articular_site_narrow_and_pointed_posteriorly

Node 131 :
All trees:
Neural_B* (117): regular,_often_hexagonal,_longer_than_wide --> irregular_in_shape,_wider_than_long
Vertebral_C (129): on_neural_V --> sulcus_between_V_3_and_4_on_neural_VI
Posterior_plastral_fontanelle (222): posterior_plastral_fontanella_between_the_xiphiplastra_and/or_the_hypoplastra:__absent_in_adult_stage --> retained_in_adults
Some trees:
Plastron_A (131): ligamentous --> connection_between_carapace_and_plastron_osseous

Node 132 :
All trees:
Quadrate_A (46): by_pt --> by_qu_and_pro
Quadrate_G (51): present --> processus_trochlearis_oticum_absent
Pterygoid_D (58): present --> pterygoid-basioccipital_contact_absent
Opisthotic_C (74): present,_with_an_incipient_enclosed_middle_ear_region --> ventral_ridge_on_opisthotic_absent
Xiphiplastron_A (149): distinct_anal_notch_absent --> present
Gular_A (153): one_pair --> only_one_scute
Inframarginal_A (165): present --> absent
Cervical_articulation_A (172): not_formed --> formed
Pelvis_A (204): pelvis-shell_attachment_by_ligaments --> ischium_attached_to_plastron_by_a_broad_suture
Pterygoid_extension (236): pterygoid_extending_to_posterior_end_of_skull_and_covering_prootic --> _pterygoid_not_extending_to_posterior_end_of_skull_and_covering_prootic

Node 133 :
All trees:
Parietal_H* (18): absent_or_weak,_foramen_stapedio-temporale_concealed_in_dorsal_view --> moderate,_f.s.t._but_not_entire_processes_trochlearis_exposed_in_dorsal_view
Basioccipital_A (70): with_two_or_one_ventral_basioccipital_tubercle --> tubercle_absent
Cervical_rib_A (168): present --> absent
Caudal_C* (190): anterior_caudal_vertebrae_opisthocoelous --> anterior_caudal_vertebrae_procoelous_or_platycoelous
Pubis_B* (207): Epipubis_process_osseous_or_calcified --> cartilaginous_or_absent
Tail_length (231): tail_as_long_as_carapace --> tail_clearly_shorter_than_carapace

Node 134 :
All trees:
Pterygoid_B (56): basipt_process_present_and_sutured_articulation --> basipt_process_absent_and_sutured_articulation
Nuchal_A (113): cervical_articulates_with_nuchal_along_a_blunt_facet --> articulation_absent
Scapula_A* (198): lamina_between_the_dorsal_process_of_the_scapula_and_the_acromion_reduced:_Kallokibotion --> lamina_between_the_dorsal_process_of_the_scapula_and_the_acromion_absent

Node 135 :
All trees:
Cervical_articulation_A (172): formed --> not_formed
Nuchal_emargination (230): absent_or_indistinct, or present,_excludes_peripheral_1, or deep_and_involves_peripheral_1 --> absent_or_indistinct, or present,_excludes_peripheral_1

Node 136 :
All trees:
Cranial_scutes_A* (89): present --> absent
Carapace_E* (112): like_in_trionychians --> Sculpturing_of_the_shell_like_in_Hydromedusa
Cervical_vertebra_F* (175): 4th --> 5th

Node 137 :
All trees:
Stapedial_artery_C*_ (82): Foramen_stapedio-temporalis_located_in_the_dorsal_part_of_the_otic_region_and_points_dorsally --> located_in_the_anterior_wall_of_the_otic_region_and_points_anteriorly
Costal_B (121): medial_contact_of_posterior_costals_absent --> medial_contact_of_up_to_three_posterior_costals_present
Vertebral_B (128): vertebral_II-IV_broader_than_pleurals --> vertebrals_II-IV_narrower_or_as_narrow_as_pleurals
Some trees:
Nuchal_C* (115): wider_than_long --> longer_than_wide_or_as_long_as_wide

Node 138 :
All trees:
Parietal_H* (18): strong,_entire_processus_trochlearis_exposed_in_dorsal_view --> absent_or_weak,_foramen_stapedio-temporale_concealed_in_dorsal_view
Supraoccipital_A (66): crista_occipitalis_poorly_developed --> protruding_significantly_posterior_to_the_foramen_magnum
Dentary_A (107): sutured_only --> medial_contact_of_dentaries_fused
Cervical_A (125): one_cervical_present --> cervicals_absent,_carapacial_scutes_otherwise_present
Hyoplastron_B* (145): terminates_on_peripheral_3 --> Axillary_buttress_terminates_on_peripheral_2_or_1

Node 139 :
All trees:
Costal_A (120): medial_contact_of_costal_I_absent --> present
Costal_B (121): medial_contact_of_up_to_three_posterior_costals_present --> medial_contact_of_all_costals_present

Node 140 :
All trees:
Musk_ducts_A* (119): absent --> present
Mesoplastron_A (146): 1_reduced_pair --> absent
Some trees:
Plastron_A (131): ligamentous --> connection_between_carapace_and_plastron_osseous

Node 141 :
All trees:
Frontal_B* (10): not_fused --> fused
Parietal_A (11): parietal-squamosal_contact_present --> absent
Quadrate_I* (53): Quadrate-basisphenoid_contact_absent --> present
Hypoplastron_A (147): peripheral_and_costal_V --> inguinal_buttresses_contact_peripherals_only
Extragular_B (155): medial_contact_of_extragulars_absent --> present,_contacting_one_another_anterior_to_gulars
Humerus_B* (200): shoulder_present --> shoulder_absent:_pleurodires

Node 142 :
All trees:
No synapomorphies

Node 143 :
All trees:
Nasal_A (0): present --> absent
Prefrontal_A (3): medial_contact_on_dorsal_skull_roof_absent --> medial_contact_on_dorsal_skull_roof_present
Prefrontal_D (6): reduced --> prefrontal_exposure_large
Parietal_A (11): parietal-squamosal_contact_present --> absent
Vomer_B (41): vomer-pterygoid_contact_in_palatal_view_present --> absent,_medial_contact_of_palatines_present
Quadrate_D (48): precolumellar_fossa_absent --> large_and_deep
Cervical_A (125): one_cervical_present --> cervicals_absent,_carapacial_scutes_otherwise_present
Cervical_vertebra_F* (175): 4th --> Biconvex_cervical_vertebra_in_the_middle_of_the_neck_2nd

Node 144 :
All trees:
Parietal_H* (18): strong,_entire_processus_trochlearis_exposed_in_dorsal_view --> absent_or_weak,_foramen_stapedio-temporale_concealed_in_dorsal_view
Pterygoid_K* (65): Fossa_podocnemidoidea_absent --> present
Humeral_B* (160): Humero-pectoral_sulcus_only_in_the_hyoplastra --> humero-pectoral_sulcus_crossing_the_entoplastron

Node 145 :
All trees:
Maxilla_D*_ (38): Triturating_surface_with_only_labial_ridge_present --> labial,_lingual_and_accesory_ridges_present
Musk_ducts_A* (119): absent --> present
Some trees:
Plastron_A (131): connection_between_carapace_and_plastron_osseous, or ligamentous --> connection_between_carapace_and_plastron_osseous
Plastron_B (132): present --> central_plastral_fontanella_absent

Node 146 :
All trees:
Cranial_scutes_A* (89): present --> absent

Node 147 :
All trees:
Nasal_B (1): nasals_contact_another_medially_along_their_entire_length --> medial_contact_of_nasals_partially_or_fully_hindered_by_long_anterior_fl
Pterygoid_B (56): basipt_process_absent_and_sutured_articulation --> basipt_process_present_and_sutured_articulation
Pterygoid_G (61): medial_contact_of_pterygoids_present --> absent

Node 148 :
All trees:
Parietal_A (11): parietal-squamosal_contact_present --> absent

Node 149 :
All trees:
Parietal_H* (18): absent_or_weak,_foramen_stapedio-temporale_concealed_in_dorsal_view --> moderate,_f.s.t._but_not_entire_processes_trochlearis_exposed_in_dorsal_view
Epipterygoid_A (54): present,_laminar --> absent
Basioccipital_A (70): with_two_or_one_ventral_basioccipital_tubercle --> tubercle_absent
Vertebral_B (128): vertebral_II-IV_broader_than_pleurals --> vertebrals_II-IV_narrower_or_as_narrow_as_pleurals
Some trees:
Dorsal_rib_A (184): length_first_thoracic_rib_long,_extends_full_legth_of_first_costal_and_may_contact_peripherals --> intermediate,_in_contact_with_axillary_buttresses

Node 150 :
All trees:
Prefrontal_D (6): reduced --> absent_or_near_absent
Extragular_D* (157): Only_in_the_epiplastra --> Reach_the_entoplastron

Node 151 :
All trees:
Extragular_B (155): medial_contact_of_extragulars_absent --> present,_contacting_one_another_posterior_to_gulars

Node 152 :
All trees:
Cervical_A (125): one_cervical_present --> more_than_one_cervical_present
Caudal_B (189): all_centra_amphicoelous --> formed_centra
Some trees:
Maxilla_D*_ (38): Triturating_surface_with_only_labial_ridge_present --> labial_and_lingual_ridge_present

Node 153 :
All trees:
Prefrontal_A (3): medial_contact_on_dorsal_skull_roof_absent --> medial_contact_on_dorsal_skull_roof_present
Prefrontal_D (6): reduced --> prefrontal_exposure_large
Some trees:
Parietal_C (13): elongated --> length_of_anterior_extension_of_the_lateral_braincase_wall_inter
Supraoccipital_A (66): crista_occipitalis_poorly_developed --> protruding_significantly_posterior_to_the_foramen_magnum

Node 154 :
All trees:
Hyoplastron_B* (145): terminates_on_peripheral_3 --> Axillary_buttress_terminates_on_peripheral_2_or_1
Mesoplastron_A (146): 1_reduced_pair --> absent
Carotid_canal_entry (235): fpcci_is_not_at_back_of_skull --> fpcci_located_at_back_of_skull_in_pterygoid
Some trees:
Parietal_E (15): processus_inferior_parietalis_forming_posterior_margin_for_nerv_trigemini_absent --> ..._present
Maxilla_D*_ (38): Triturating_surface_with_only_labial_ridge_present --> labial_and_lingual_ridge_present
Pterygoid_J* (64): not_reaching_the_exoccipitals --> reaching_the_exoccipitals
Nuchal_emargination (230): absent_or_indistinct --> present,_excludes_peripheral_1

Node 155 :
Some trees:
Parietal_E (15): ..._present --> processus_inferior_parietalis_forming_posterior_margin_for_nerv_trigemini_absent
Maxilla_D*_ (38): labial_and_lingual_ridge_present --> Triturating_surface_with_only_labial_ridge_present
Opisthotic_C (74): present,_with_an_incipient_enclosed_middle_ear_region --> present,_but_modified_with_a_enclosed_middle_ear_region
Hyoplastron_A (143): peripherals_and_first_costal --> axillary_buttresses_contact_peripherals_only
Hypoplastron_A (147): peripheral_and_costal_V --> inguinal_buttresses_contact_peripherals_only

Node 156 :
All trees:
Parietal_H* (18): strong,_entire_processus_trochlearis_exposed_in_dorsal_view --> moderate,_f.s.t._but_not_entire_processes_trochlearis_exposed_in_dorsal_view
Squamosal_E*_ (28): Qu-Sq_contact_tightly_sutured --> wide_open
Nuchal_A (113): articulation_absent --> cervical_articulates_with_nuchal_along_a_raised_pedestal
Costal_C (122): absent,_costals_fully_or_almost_fully_ossified,_fontanelles_abs_or_red --> present
Plastron_B (132): central_plastral_fontanella_absent --> present
Xiphiplastron_B (150): xiphiplastra_narrow_absent --> present
Manus_B (216): paddles_absent --> short_paddles_present
Nuchal_emargination (230): absent_or_indistinct --> present,_excludes_peripheral_1, or deep_and_involves_peripheral_1

Node 157 :
All trees:
Cruciform_plastron (232): absent --> present

Node 158 :
All trees:
Hyoplastron_B* (145): Axillary_buttress_terminates_on_peripheral_2_or_1 --> terminates_on_peripheral_3
Extragular_A (154): present --> absent
Humerus_A* (199): Ectepicondylar_foramen_in_a_channel --> only_a_groove

Node 159 :
All trees:
Nasal_A (0): present --> absent
Parietal_A (11): parietal-squamosal_contact_present --> absent
Pterygoid_H (62): pterygoid_contribution_to_foramen_palatinum_posterius_present --> absent
Hypoplastron_B* (148): Inguinal_buttress_terminates_on_peripheral_8 --> 7
Humeral_B* (160): Humero-pectoral_sulcus_only_in_the_hyoplastra --> humero-pectoral_sulcus_crossing_the_entoplastron
Cervical_articulation_J* (178): double_articulation_between_6th_and_7th_absent --> present
Cervical_articulation_L* (180): double_articulation_between_7th_and_8th_absent --> present
Dorsal_rib_A (184): length_first_thoracic_rib_long,_extends_full_legth_of_first_costal_and_may_contact_peripherals --> intermediate_to_short
Dorsal_vertebra_A (187): anterior_articulation_of_first_dorsal_centrum_faces_at_most_slightly_anteroventrally --> faces_strongly_anteroventrally
Humerus_D* (202): lateral_process_seen_in_dorsal_view --> lateral_process_not_seen_in_dorsal_view
Nuchal_emargination (230): present,_excludes_peripheral_1 --> absent_or_indistinct
Articulation_of_posterior_cervical_centra (233): circular_or_subcircular_outline --> greatly_flattened_outline

Node 160 :
All trees:
Parietal_H* (18): moderate,_f.s.t._but_not_entire_processes_trochlearis_exposed_in_dorsal_view --> strong,_entire_processus_trochlearis_exposed_in_dorsal_view
Vertebral_B (128): vertebral_II-IV_broader_than_pleurals --> vertebrals_II-IV_narrower_or_as_narrow_as_pleurals
Cervical_vertebra_B (170): ventral_keels_absent_or_slightly_developed_in_all_vertebrae --> ventral_keels_more_developed_on_posterior_vertebrae
Cervical_articulation_A (172): not_formed --> formed
Some trees:
Parietal_C (13): length_of_anterior_extension_of_the_lateral_braincase_wall_inter --> elongated
Plastron_B (132): present --> central_plastral_fontanella_absent

Node 161 :
All trees:
Premaxilla_C (33): foramen_praepalatinum_present --> absent,_premaxillae_well-ossified
Maxilla_C*_ (37): Secondary_palate_formed_by_premaxilla,_maxilla,_and_vomer,_palatines_not_contacting_in_midline_absent --> formed_by_premaxilla,_maxilla,_and_vomer,_palatines_not_contacting_in_midline_present
Vomer_D (43): vomer-premaxilla_contact_present --> absent

Node 162 :
All trees:
Parietal_A (11): absent --> parietal-squamosal_contact_present
Parietal_H* (18): moderate,_f.s.t._but_not_entire_processes_trochlearis_exposed_in_dorsal_view --> absent_or_weak,_foramen_stapedio-temporale_concealed_in_dorsal_view
Antrum_postoticum_A (49): fully_developed --> incipient

Pterygoid_F (60): foramen_palatinum_posterius_present --> absent
Basisphenoid_A (76): rostrum_basisphenoidale_flat --> rod-like,_thick,_and_rounded
Manus_B (216): short_paddles_present --> elongate_paddles_present

Node 163 :
All trees:
Carapace_A (108): carapacial_scutes_present --> partially_present
Plastral_scutes_A (151): present --> absent
Cervical_vertebra_H* (181): total_height_of_centra_and_neural_arch_much_shorter_than_the_anteroposterior_length_of_the_cervical_centra_ --> total_height_of_centra_and_neural_arch_longer_than_the_anteroposterior_length_of_the_cervical_centra_

Node 164 :
All trees:
Premaxilla_E (35): distinct,_medial_premaxillary_hook_along_the_labial_margin_absent --> present
Some trees:
Plastron_B (132): central_plastral_fontanella_absent --> present
Posterior_plastral_fontanelle (222): posterior_plastral_fontanella_between_the_xiphiplastra_and/or_the_hypoplastra:__absent_in_adult_stage --> retained_in_adults

Node 165 :
All trees:
Frontal_A (9): present --> frontal_contribution_to_orbit_absent
Quadrate_F:_incisura_columella_auris (50): present,_but_qu_and_the_op_for_an_angle_less_90_degrees_in_lat_view --> present_and_closed,_but_only_enclosing_the_stapes
Anal_A (164): only_cover_parts_of_the_xiphiplastra --> anteromedially_overlap_onto_hypoplastra

Node 166 :
Some trees:
Abdominal_A (163): present,_with_medial_contact --> present,_medial_contact_absent

Node 167 :
All trees:
No synapomorphies

Node 168 :
All trees:
Xiphiplastron_A (149): distinct_anal_notch_absent --> present
Cervical_vertebra_G* (176): Biconcave_cervical_vertebra_absent --> present

Node 169 :
All trees:
Plastron_A (131): ligamentous --> connection_between_carapace_and_plastron_osseous
Hyoplastron_A (143): axillary_buttresses_contact_peripherals_only --> peripherals_and_first_costal
Hypoplastron_A (147): inguinal_buttresses_contact_peripherals_only --> peripheral_and_costal_V
Hypoplastron_B* (148): 7 --> Inguinal_buttress_terminates_on_peripheral_8

Node 170 :
All trees:
Pterygoid_J* (64): reaching_the_exoccipitals --> not_reaching_the_exoccipitals
Suprapygal_A* (124): two_elements --> one_element

Node 171 :
All trees:
Costal_D* (123): absence_of_alternative_short_and_long_ends_in_the_lateral_part_of_the_costals --> presence
Hyoplastron_B* (145): terminates_on_peripheral_3 --> Axillary_buttress_terminates_on_peripheral_2_or_1
Pes_C* (220): 5_digits --> 4_digits

Node 172 :
All trees:
Humeral_B* (160): humero-pectoral_sulcus_crossing_the_entoplastron --> Humero-pectoral_sulcus_only_in_the_hyoplastra
Manus_A (215): most_digits_with_three_elongate_phalanges --> most_digits_with_two_shortened_phalanges

Node 173 :
All trees:

Pectoral_B* (162): antero-posteriorly_developed --> very_short_antero-posteriorly

Node 174 :
All trees:
Plastron_A (131): ligamentous --> connection_between_carapace_and_plastron_osseous
Hyoplastron_A (143): axillary_buttresses_contact_peripherals_only --> peripherals_and_first_costal
Hypoplastron_A (147): inguinal_buttresses_contact_peripherals_only --> peripheral_and_costal_V
Inframarginal_B* (166): 3_or_more --> 2

Node 175 :
All trees:
No synapomorphies

Node 176 :
All trees:
Cervical_A (125): one_cervical_present --> cervicals_absent,_carapacial_scutes_otherwise_present

Node 177 :
All trees:
Squamosal_A (24): squamosal-postorbital_contact_present --> absent
Maxilla_D*_ (38): Triturating_surface_with_only_labial_ridge_present --> labial,_lingual_and_accesory_ridges_present
Inframarginal_B* (166): 3_or_more --> 2
Cervical_articulation_I* (177): double_articulation_between_5th_and_6th_absent --> present

Node 178 :
All trees:
Squamosal_A (24): squamosal-postorbital_contact_present --> absent
Palatine_A (45): palatine_contribution_to_anterior_extension_of_lat_braincase_absent --> present,_well-developed
Carapace_B (109): tricarinate_carapace_absent --> present,_but_only_slightly

Node 179 :
All trees:
Maxilla_D*_ (38): Triturating_surface_with_only_labial_ridge_present --> labial,_lingual_and_accesory_ridges_present
Stapedial_artery_B (81): significantly_reduced_in_size --> absent
Hyoplastron_A (143): axillary_buttresses_contact_peripherals_only --> peripherals_and_first_costal
Some trees:
Nuchal_B (114): present,_process_crosses_peripheral_I_to_contact_pe_II_ --> elongate_costiform_process_of_nuchal_absent
Abdominal_A (163): present,_medial_contact_absent --> present,_with_medial_contact

Node 180 :
All trees:
Plastron_A (131): ligamentous --> connection_between_carapace_and_plastron_osseous
Cruciform_plastron (232): present --> absent
Some trees:
Stapedial_artery_B (81): relatively_large --> significantly_reduced_in_size

Node 181 :
All trees:
Peripheral_A (118): 11_pairs --> 10_pairs
Hyoplastron_B* (145): terminates_on_peripheral_3 --> terminates_on_peripheral_4
Abdominal_A (163): present,_medial_contact_absent --> absent
Inframarginal_B* (166): 3_or_more --> 2
Some trees:
Frontal_A (9): present --> frontal_contribution_to_orbit_absent
Parietal_F* (16): not_contribute_to_the_processus_trochlearis_oticum --> contributes_to_the_processus_trochlearis_oticum
Quadratojugal_B (22): quadratojugal-maxilla_contact_absent --> present
Quadrate_H*_ (52): Processus_trochlearis_oticum_formed_by_a_great_contribution_of_quadrate --> small_contribution_of_the_quadrate
Costal_B (121): medial_contact_of_posterior_costals_absent --> medial_contact_of_up_to_three_posterior_costals_present
Inframarginal_C* (167): axillar_and_inguinal_not_in_contact --> axillar_and_inguinal_in_contact
Dorsal_rib_B (185): contact_dorsal_rib_9-10_with_costals_present --> absent

Node 182 :
All trees:

Costal_A (120): medial_contact_of_costal_I_absent --> present
Entoplastron_E (140): present --> absent
Intergular_A (158): absent --> present
Humeral_A (159): 1_pair --> 2_pair_subdivided_by_a_plastral_hinge

Node 183 :
All trees:
Nuchal_C* (115): wider_than_long --> longer_than_wide_or_as_long_as_wide

Node 184 :
All trees:
Neural_A (116): neural_formula_6>4<6<6<6<6_absent --> present
Plastron_A (131): ligamentous --> connection_between_carapace_and_plastron_osseous
Carotid_canal_split (237): 3 -->
not_enclosed_but_carotid_canal_is_covered_ventrally_from_the_posterior_end_of_the_skull

Node 185 :
All trees:
Carapace_D_* (111): Sculpturing_of_the_shell_absent --> present

Node 186 :
All trees:
Costal_B (121): medial_contact_of_posterior_costals_absent -->
medial_contact_of_up_to_three_posterior_costals_present
Suprapygal_A* (124): two_elements --> one_element

Node 187 :
All trees:
Peripheral_gutter (228): peripheral_gutter_absent_or_only_anteriorly_developed -->
peripheral_gutter_extensively_developed_along_anterior_and_bridge_peripherals

Node 188 :
All trees:
No synapomorphies

Node 189 :
All trees:
Jugal_B (20): jugal_participation_to_upper_temporal_rim_absent --> present
Carapace_A (108): partially_present --> absent
Peripheral_A (118): 10_pairs --> less_than_10_pairs
Posterior_plastral_fontanelle (222):
posterior_plastral_fontanella_between_the_xiphiplastra_and/or_the_hypoplastra:__absent_in_adult_stage -->
retained_in_adults
Some trees:
Premaxilla_D (34): exclusion_of_premaxilla_from_the_apertura_narium_externa_absent --> present
Maxilla_A* (36): do_not_contact_each_other_in_ventral_view --> contacts_each_other_in_ventral_view
Entoplastron_D (139): entoplastron_V-shaped_absent --> present
Cervical_articulation_H (173): 8)dorsal --> none,_vertebrae_only_meet_at_zygapophyses
Humerus_A* (199): Ectepicondylar_foramen_in_a_channel --> only_a_groove
Humerus_B* (200): shoulder_present --> shoulder_absent:_pleurodires
Manus_and_Pes_B* (221): Hyperphalangy_manus_digits_4_and_5,_pes_digit_4_no --> yes

Node 190 :
All trees:
Prefrontal_C (5): prefrontal-palatine_contact_present --> prefrontal-palatine_contact_absent
Premaxilla_B (32): fusion_of_premaxilla_absent --> present
Premaxilla_C (33): foramen_praepalatinum_present --> absent,_foramen_intermaxillaris_present
Vomer_B (41): vomer-pterygoid_contact_in_palatal_view_present --> absent,_medial_contact_of_palatines_present
Vomer_D (43): vomer-premaxilla_contact_present --> absent
Pterygoid_G (61): medial_contact_of_pterygoids_present --> absent
Carapace_A (108): carapacial_scutes_present --> partially_present
Peripheral_A (118): 11_pairs --> 10_pairs
Plastral_scutes_A (151): present --> absent
Cervical_vertebra_B (170): ventral_keels_more_developed_on_posterior_vertebrae -->
ventral_keels_absent_or_slightly_developed_in_all_vertebrae
Pubis_A* (206): lateral_process_small,_poorly_developed,_columnar --> lateral_process_well_developed_and_flat
Manus_C (217): flippers_absent --> short_flippers_present, or elongate_flippers_present

Nuchal_posterior_edge (234): less_than_3_times_longer_than_the_lateral_edge --> more_than_3_times_longer
Some trees:
Hyoplastron_B* (145): Axillary_buttress_terminates_on_peripheral_2_or_1 --> terminates_on_peripheral_4

Node 191 :
All trees:
Marginal_A* (130): marginal_scales_overlap_onto_costals_absent --> present

Node 192 :
All trees:
Plastral_scutes_B (152): pronounced_midline_plastral_sulcus_sinuous_absent --> present

Node 193 :
All trees:
Pterygoid_B (56): basipt_process_absent_and_sutured_articulation --> basipt_process_present_and_sutured_articulation
Basioccipital_A (70): tubercle_absent --> with_two_or_one_ventral_basioccipital_tubercle
Musk_ducts_A* (119): absent --> present
Anal_A (164): only_cover_parts_of_the_xiphiplastra --> anteromedially_overlap_onto_hypoplastra
Cervical_rib_A (168): absent --> present
Peripheral_gutter (228): peripheral_gutter_absent_or_only_anteriorly_developed --> peripheral_gutter_extensively_developed_along_anterior_and_bridge_peripherals
Tail_length (231): tail_clearly_shorter_than_carapace --> tail_as_long_as_carapace
Carotid_canal_split (237): 3 --> not_enclosed_but_carotid_canal_is_covered_ventrally_from_the_posterior_end_of_the_skull

Node 194 :
All trees:
Nuchal_emargination (230): present,_excludes_peripheral_1 --> absent_or_indistinct

Node 195 :
All trees:
Parietal_H* (18): strong,_entire_processus_trochlearis_exposed_in_dorsal_view --> moderate,_f.s.t._but_not_entire_processes_trochlearis_exposed_in_dorsal_view

Node 196 :
All trees:
Basisphenoid_B (77): paired_pits_on_ventral_surface_absent --> present
Epiplastron_A (141): epiplastra_and_entoplastron_narrow_and_elongate_absent --> present
Cruciform_plastron (232): absent --> present

Node 197 :
All trees:
Cervical_A (125): one_cervical_present --> cervicals_absent,_carapacial_scutes_otherwise_present
Neural_number (223): less_than_9_elements --> 9_elements
First_vertebral (227): vertebral_1_does_not_enter_anterior_margin_of_carapace --> enters_anterior_margin
Peripheral_gutter (228): peripheral_gutter_extensively_developed_along_anterior_and_bridge_peripherals --> peripheral_gutter_absent_or_only_anteriorly_developed
Nuchal_emargination (230): present,_excludes_peripheral_1 --> deep_and_involves_peripheral_1
Nuchal_posterior_edge (234): less_than_3_times_longer_than_the_lateral_edge --> more_than_3_times_longer

Node 198 :
All trees:
Cervical_vertebra_E* (174): present --> Biconvex_cervical_vertebra_in_the_middle_of_the_neck_absent

Node 199 :
All trees:
Plastron_B (132): central_plastral_fontanella_absent --> present

Node 200 :
All trees:
Costal_rib_distal_end (229): distal_end_of_dorsal_rib_not_visible_or_only_within_costo-peripheral_fontanelles_on_the_dorsal_face_of_the_carapace --> costo-peripheral_fontanelles_absent,_distal_end_of_posterior_dorsal_ribs_visible_or_distal_end_of_posterior_costals_narrow_and_surrounded_by_the_peripheral

Node 201 :
All trees:

Vertebral_B (128): vertebrals_II-IV_narrower_or_as_narrow_as_pleurals --> vertebral_II-IV_broader_than_pleurals
Posterior_plastral_fontanelle (222): posterior_plastral_fontanella_between_the_xiphiplastra_and/or_the_hypoplastra:__absent_in_adult_stage --> retained_in_adults

Node 202 :
All trees:
Pterygoid_D (58): present --> pterygoid-basioccipital_contact_absent
Pterygoid_J* (64): reaching_the_exoccipitals --> not_reaching_the_exoccipitals
Some trees:
Quadrate_D (48): precolumellar_fossa_absent --> large_and_deep
Epiplastron_A (141): present --> epiplastra_and_entoplastron_narrow_and_elongate_absent

Node 203 :
All trees:
Epiplastron_A (141): epiplastra_and_entoplastron_narrow_and_elongate_absent --> present
Extragular_B (155): medial_contact_of_extragulars_absent --> present,_contacting_one_another_anterior_to_gulars
Nuchal_emargination (230): absent_or_indistinct, or present,_excludes_peripheral_1, or deep_and_involves_peripheral_1 --> broad,_involved_peripheral_II
Some trees:
Frontal_A (9): present --> frontal_contribution_to_orbit_absent
Basisphenoid_B (77): paired_pits_on_ventral_surface_absent --> present
Cranial_scute_C* (91): yes --> Scute_X_much_smaller_than_D_scute_no
Antrum_postoticum (238): region_of_antrum_postoticum_enlarged_and_laterally_enclosed --> region_of_antrum_postoticum_enlarged,_but_not_enclosed_laterally
Jugal/quadrate_contact (239): jugal_clearly_not_in_contact_with_quadrate --> jugal_nearly_or_clearly_in_contact_with_quadrate
Parabasisphenoid_decorated_by_ridges (240): absent --> present

Node 204 :
All trees:
Opisthotic_C (74): present,_with_an_incipient_enclosed_middle_ear_region --> ventral_ridge_on_opisthotic_absent
Recessus_scalae_tympani_A* (83): well_developed --> almost_inexistent,_not_surrounded_by_bone
Carapace_D_* (111): Sculpturing_of_the_shell_absent --> present
Entoplastral_scute (241): absent --> present
Secondary_pair_of_basioccipital_tubercles_formed_by_pterygoid (242): absent --> present
Shell_covered_by_highly_distinct_turbercles (243): absent --> present

C. Alternative positions of wildcard taxa

a: *Heckerochelys romani*, **b:** *Eileanchelys waldmani*, **c:** *Indochelys spatulata,* **d:** *Patagoniaemys gasparinae,* **e:** *Xinjiangchelys junggarensis*

Strict consensus of 550 trees

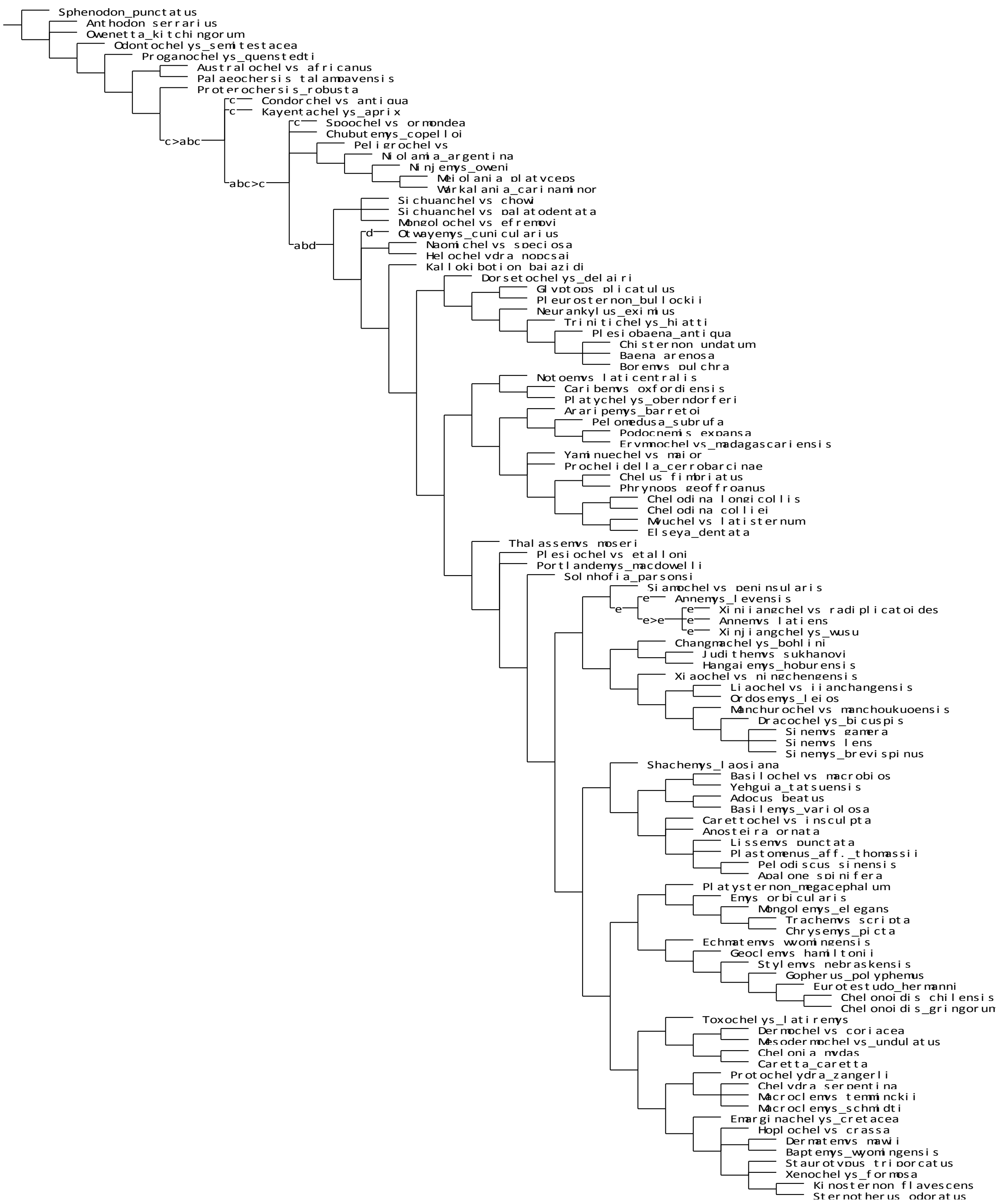

D. Frequency differences values of standard bootstrap resampling of 1000 replicates

GC values, 1000 replicates, cut=1 (tree 0) - Standard Bootstrap

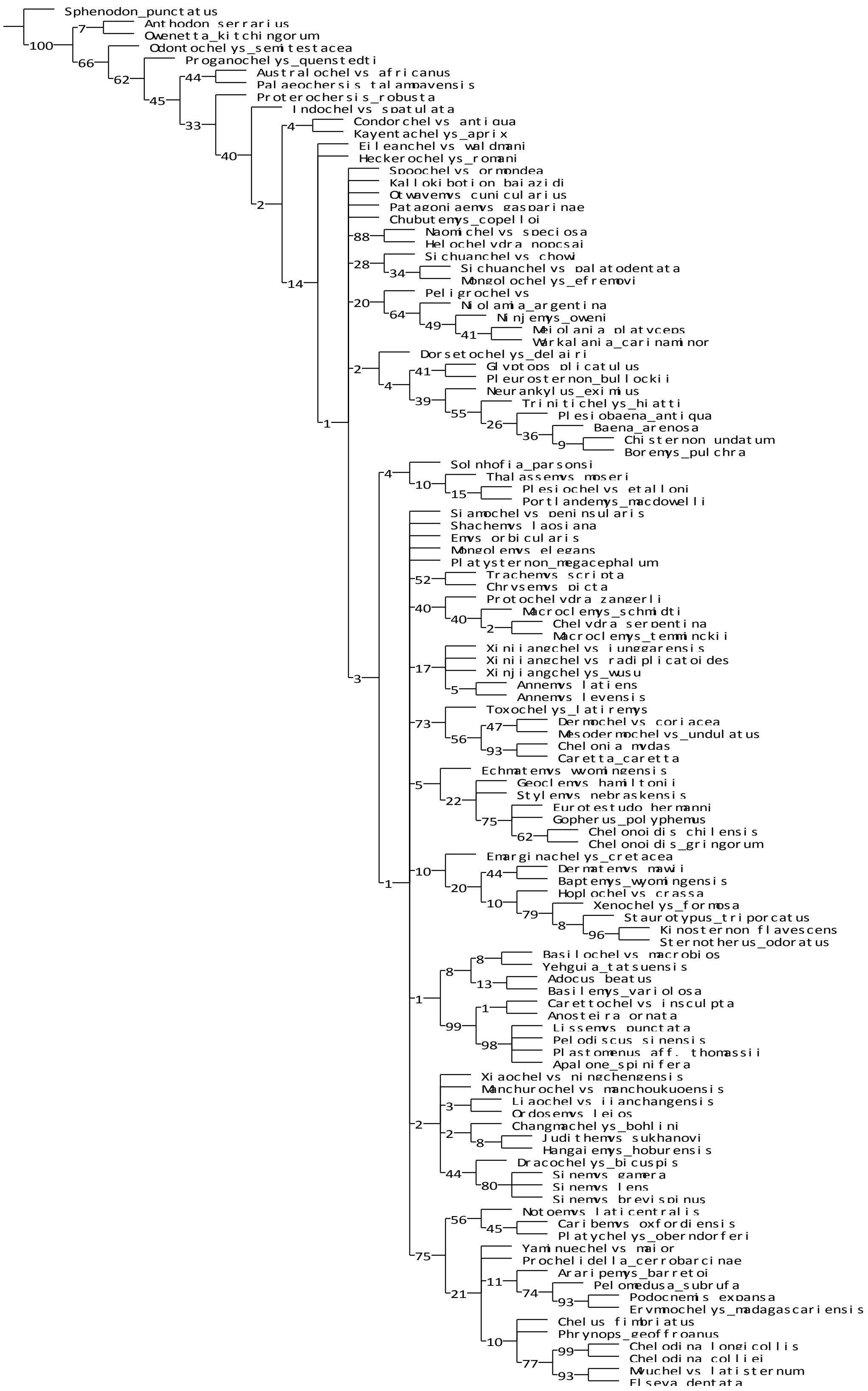

E. Frequency differences values of standard bootstrap resampling of 1000 replicates with 5 wild card taxa excluded

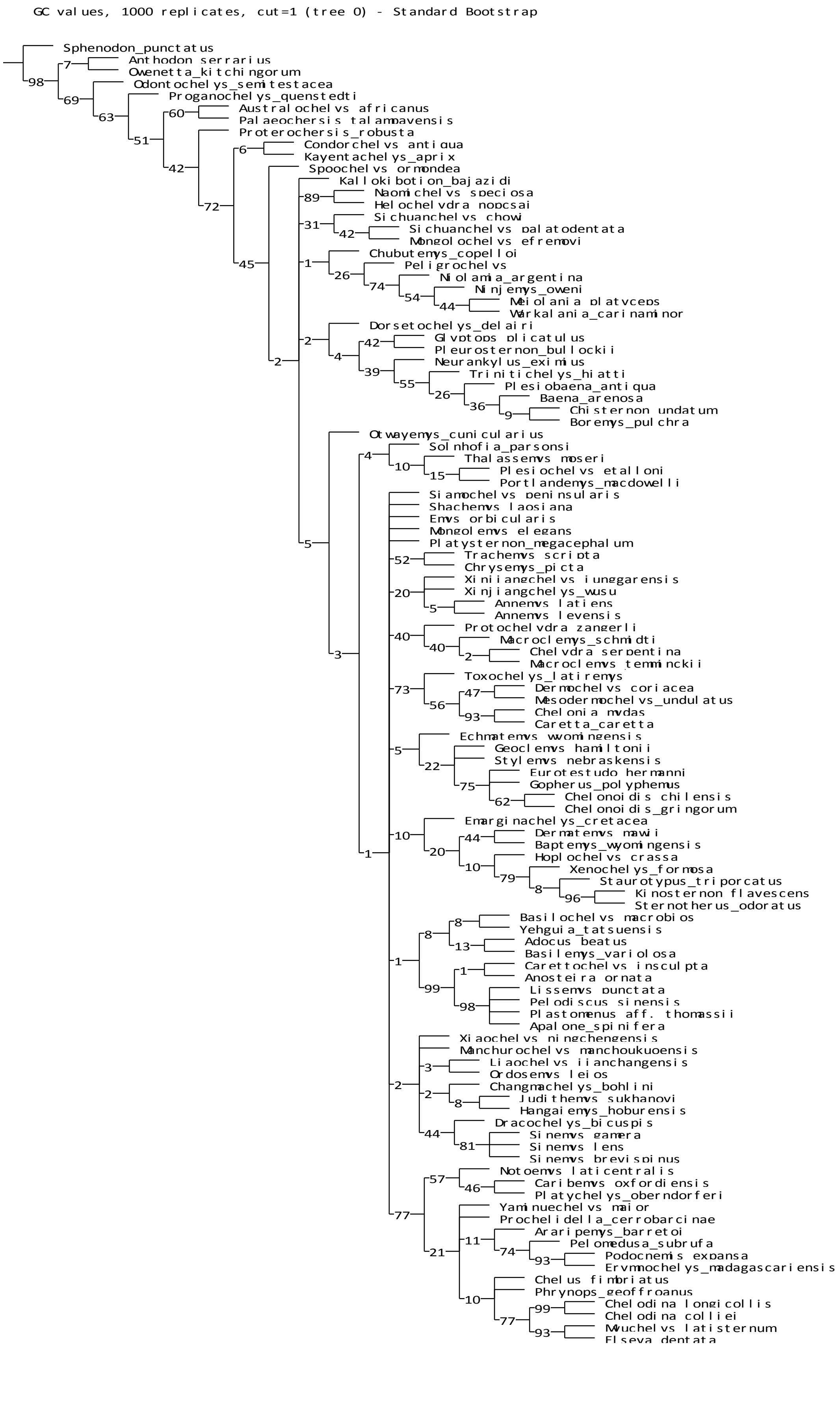

Supplement: Additional file 3: — Results of the phylogenetic analysis. (PDF 392 kb) [file 12862_2016_762_MOESM3_ESM.pdf]
